# Structure-Based Discovery of Lipoteichoic Acid Synthase Inhibitors

**DOI:** 10.1021/acs.jcim.2c00300

**Published:** 2022-05-09

**Authors:** Xavier Chee Wezen, Aneesh Chandran, Rohan Sakariah Eapen, Elaine Waters, Laura Bricio-Moreno, Tommaso Tosi, Stephen Dolan, Charlotte Millership, Aras Kadioglu, Angelika Gründling, Laura S. Itzhaki, Martin Welch, Taufiq Rahman

**Affiliations:** † Science Program, School of Chemical Engineering and Science, Faculty of Engineering, Computing and Science, Swinburne University of Technology Sarawak, Kuching 93350, Malaysia; ‡ Department of Biotechnology & Microbiology, Kannur University, Kannur 670 661, Kerala, India; § Department of Clinical Infection Microbiology and Immunology, Institute of Infection and Global Health, University of Liverpool, Liverpool L69 7BE, U.K.; ∥ Section of Molecular Microbiology and MRC Centre for Molecular Bacteriology and Infection, Imperial College London, London SW7 2AZ, U.K.; ⊥ Department of Biochemistry, University of Cambridge, Cambridge CB2 1QW, U.K.; ¶ Department of PharmacologyUniversity of CambridgeCambridgeCB2 1PDU.K.

## Abstract

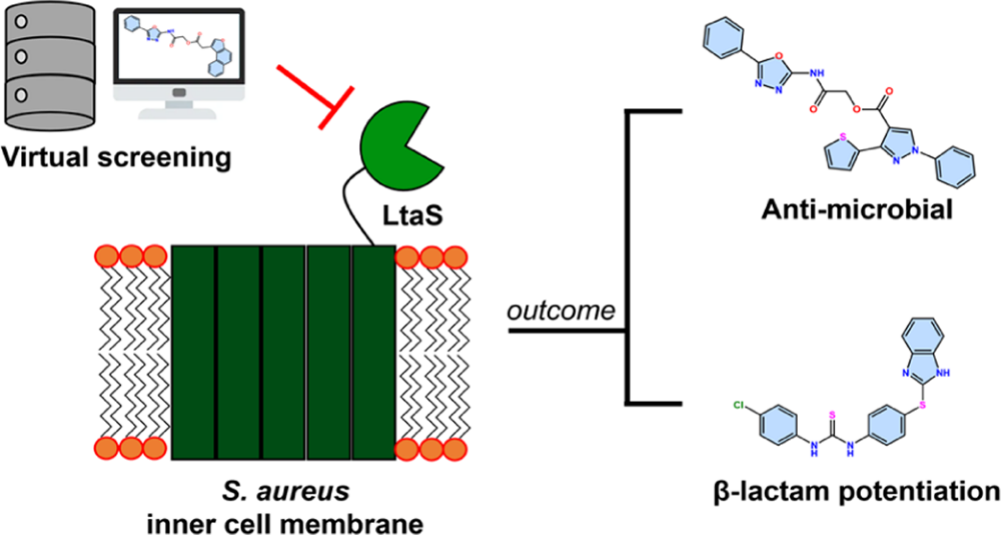

Lipoteichoic acid synthase (LtaS) is a key enzyme for the cell wall biosynthesis of Gram-positive bacteria. Gram-positive bacteria that lack lipoteichoic acid (LTA) exhibit impaired cell division and growth defects. Thus, LtaS appears to be an attractive antimicrobial target. The pharmacology around LtaS remains largely unexplored with only two small-molecule LtaS inhibitors reported, namely “compound **1771**” and the Congo red dye. Structure-based drug discovery efforts against LtaS remain unattempted due to the lack of an inhibitor-bound structure of LtaS. To address this, we combined the use of a molecular docking technique with molecular dynamics (MD) simulations to model a plausible binding mode of compound **1771** to the extracellular catalytic domain of LtaS (eLtaS). The model was validated using alanine mutagenesis studies combined with isothermal titration calorimetry. Additionally, lead optimization driven by our computational model resulted in an improved version of compound **1771**, namely, compound **4** which showed greater affinity for binding to eLtaS than compound **1771** in biophysical assays. Compound **4** reduced LTA production in *S. aureus* dose-dependently, induced aberrant morphology as seen for LTA-deficient bacteria, and significantly reduced bacteria titers in the lung of mice infected with *S. aureus*. Analysis of our MD simulation trajectories revealed the possible formation of a transient cryptic pocket in eLtaS. Virtual screening (VS) against the cryptic pocket led to the identification of a new class of inhibitors that could potentiate β-lactams against methicillin-resistant *S. aureus*. Our overall workflow and data should encourage further drug design campaign against LtaS. Finally, our work reinforces the importance of considering protein conformational flexibility to a successful VS endeavor.

*Staphyloccocus aureus* is an opportunistic pathogen that can cause mild to serious infections including skin and soft-tissue infections, endocarditis, osteomyelitis, and meningitis.^1−3^ Healthcare-associated methicillin-resistant *S. aureus* (MRSA) remains a key nosocomial pathogen in which resistance to all licensed antistaphylococcal drugs has been reported.^4^ In recent years, the emergence of community-associated MRSA has resulted in an increase in infections and presents a formidable challenge for infection management worldwide.^5^ In fact, it has been reported that MRSA causes approximately 19,000 deaths in the United States annually, which is a similar figure of the combined deaths from AIDS, tuberculosis, and viral hepatitis.^6,7^ Considering this as a severe threat to public health, the World Health Organization (WHO) has listed MRSA as one of the “high priority pathogens” to encourage more research and development of novel and more efficacious therapeutics against *S. aureus* infections.

In recent times, the teichoic acid biosynthesis pathway has emerged as an attractive antibacterial target toward combating infections by Gram-positive pathogens. Teichoic acids are anionic alditol-phosphate polymers that are found in abundance within the cell envelope of Gram-positive bacteria.^8^ They are important in bacterial cell physiology and virulence and can be subcategorized into wall teichoic acid^9^ (WTA) and lipoteichoic acid^10^ (LTA). These two cell wall polymers are involved in an array of biological functions such as ion homeostasis,^11,12^ cell division,^13,14^ host immune evasion,^15^ and resistance against cationic antimicrobial peptides (e.g., polymyxin B).^16,17^ Although WTA is dispensable for cell growth and viability, WTA-null mutants show attenuated virulence and host colonization during infection.^18−20^ Additionally, MRSA strains that lack WTA are resensitized to β-lactam antibiotics.^21,22^ LTA, on the other hand, is important for bacterial survival and regulates cell division by directing the FtsZ cell division initiator protein and other autolysins.^13,23,24^ To date, several WTA inhibitors such as tunicamycin,^25^ targocil,^26^ tarocins A and B,^27^ and derivatives of ticlopidine^28^ and targocil-II^29^ have been discovered. In contrast, only two LTA synthesis inhibitors^30^ (“compound **1771**”, hereafter designated “**1771**”, and the dye Congo red^31^) are known to date. However, the carcinogenicity of Congo red limits its potential as an antibiotic. In their earlier work, Richter et al. have shown that **1771** is able to suppress LTA synthesis by inhibiting the LTA synthase (LtaS), which is a critical protein required in the LTA biosynthesis pathway.^30^ When tested in a lethal sepsis mouse model, **1771** could temporarily prolong the survival of the infected mice but lost activity over time due to in vivo instability.^30^ Nevertheless, the discovery of **1771** serves as a proof-of-principle that LtaS is druggable and can be targeted by small-molecule inhibitors. Hence, we need to identify new chemotypes of LtaS inhibitors that could potentially pave the way for developing new-generation antibiotics against Gram-positive infections including those caused by MRSA.

However, conducting a high-throughput screening (HTS) campaign to discover LtaS inhibitors can be costly and is not feasible without access to small-molecule libraries. An HTS campaign in this context will typically require screening millions of compounds for LTA synthesis inhibition, a process that can be time-consuming and expensive. In addition, validating positive “hits” from HTS can often be complicated due to the low signal-to-noise ratio of the assays used.^32^ In this regard, virtual screening (VS) is a cost-effective and suitable alternative to HTS. In VS, computational algorithms are exploited to screen large compound libraries to identify a subset of potentially active ligands (“hits”) against a target,^33,34^ and these hits then can be subjected to experimental validation. In structure-based VS, ligands are docked onto the target protein structure and their poses are scored based on their complementarity with the binding site. The VS process requires more knowledge input and thus the hit rate can be better than conventional HTS.^35^ However, accounting for protein flexibility upon ligand binding is a challenge for VS. This is because a single-crystal structure only represents a static snapshot of the protein trapped in a low-energy conformation during the crystallization process. Its adopted conformation may be irrelevant for ligand binding, especially if the ligand-binding process entails significant rearrangements in the protein backbone or side chain orientations. Although several methods have been used to circumvent this problem, the most practical solution by far is to use “ensemble docking”.^36−38^ The ensemble docking process involves sequentially docking and scoring each ligand into a set of different conformations of the target protein. In this way, the docking algorithm is able to sample multiple protein conformations to select for the one that best fits the ligands. These multiple protein conformations can be obtained from either nuclear magnetic resonance (NMR) structures or powerful computational tools such as molecular dynamics (MD) simulations.

In this work, we applied VS approaches into three aspects of our drug discovery process: (a) binding-site identification, (b) optimization of existing inhibitor, and (c) discovery of new hits. Through a systematic use of different computational approaches, we modeled a plausible inhibitor-bound LtaS complex. The structural insights derived from this model enabled us to optimize **1771** into an improved LtaS inhibitor and in discovering new chemotypes against LtaS.

## Results

### Computational Prediction of Possible Ligand-Binding Pockets of LtaS

**Figure 1:**
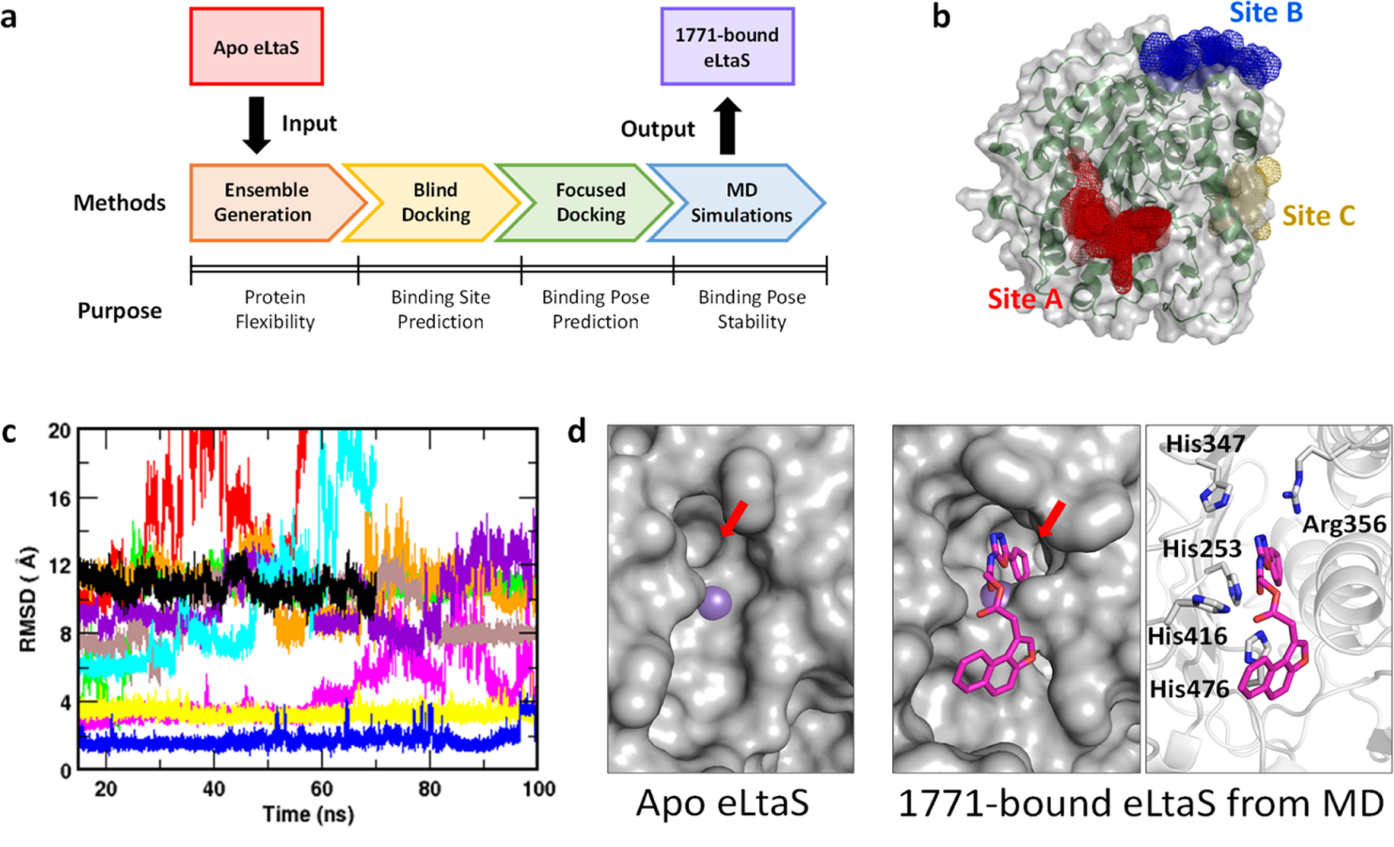
Modeling of a **1771**-bound eLtaS complex. (a) A step-wise computational approach is applied to model a **1771**-bound eLtaS complex. (b) Predicted binding sites of **1771** at eLtaS from “blind” docking studies. (c) The RMSD of the 10 **1771** poses along the simulation time with respect to their original docked positions. The poses are colored as following: pose 1, black; pose 2, red; pose 3, green; pose 4, blue; pose 5, orange; pose 6, magenta; pose 7, brown; pose 8, violet; pose 9, cyan; and pose 10, yellow. (d) Protein surface topology of the eLtaS crystal structure and the **1771**-bound protein model. Compound **1771** (magenta stick) binds in a subpocket (red arrow) formed from the conformational rotation of His253. The eLtaS active site is shown in gray surface and ribbon representation. Residues implicated in binding are shown in sticks.

LtaS is a transmembrane protein with a large extracellular catalytic domain (denoted as eLtaS).^39^ Although Richter et al. have shown that **1771** interacts with eLtaS, the precise binding mode of **1771** to eLtaS remains unknown. To rationally develop novel chemical scaffolds that could inhibit the enzyme, we attempted to uncover the structural details underlying the detailed mechanism of action of **1771**. We initially attempted cocrystallization of eLtaS with **1771** but, despite multiple crystallization trials, we were unable to obtain a ligand-bound eLtaS crystal structure (data not shown). Hence, to circumvent this problem, we decided to develop a plausible computational model of the **1771**-bound eLtaS complex (Figure 1a).

In order to capture the intrinsic protein flexibility for subsequent in silico screening, we conducted a 100 ns MD simulation of the *S. aureus* apo eLtaS (PDB-ID 2W5Q).^40^ The time-dependent root mean square deviation (RMSD) of the eLtaS protein backbone indicated that the MD-generated eLtaS conformers deviated by ∼1.7 Å from the original crystal architecture (Figure S1a). This suggested that the apo eLtaS had explored an ensemble of dynamically different conformations along the simulation time. To evaluate the extent of conformational sampling during simulation, all eLtaS conformers were projected onto a 2D plane defined by the top two principal components (PCs) obtained by PC analysis (PCA; Figure S1b). Consistent with the protein backbone RMSD analysis, the PCA also indicated that distinct conformations of apo eLtaS structures were sampled across the MD simulation timeframe. Moreover, the 2D plot shows that the conformations of the apo- and glycerolphosphate (GroP)-bound crystal structures (PDB-ID 2W5Q and 2W5T, respectively) were also sampled during the course of simulation.

After the MD simulation, all the eLtaS conformers were clustered using an RMSD-based clustering algorithm. From there, 19 distinct protein conformers were obtained to form our eLtaS ensemble (Figure S2), and these conformers were subsequently subjected to an unbiased (“blind”) docking protocol. The “blind” docking studies indicated two possible binding sites for **1771**, denoted site A and site B (Figure 1b). Site A corresponds to the catalytic site, where the substrate of eLtaS (i.e., phosphatidylglycerol; PG) binds. Meanwhile, site B lies in close proximity to some residues that are predicted to form contacts with the transmembrane region of full-length LtaS.^41^ Notably, there was seemingly an additional site (site C) that was only detected in the crystal structure, which could suggest that this site is an artifact derived from crystal packing. Out of the 2000 poses retrieved from the “blind” ensemble docking, 65% of the **1771** docked poses clustered around site A, whereas only 29% of the poses were found at site B (Table S1). Interestingly, this result corroborates with the suggestion by Richter et al. that **1771** may act as a substrate-mimetic of eLtaS. On this basis, we suggest that **1771** binds to the active site of eLtaS.

### Prediction of Possible Binding Pose of **1771**

The atomistic details of ligand–protein interactions are important to guide structure-based drug design. Hence, to obtain a reliable prediction of the **1771** binding mode, we conducted an ensemble docking study of **1771**, focusing on the active site of eLtaS. Our docking study generated 200 poses, which were subsequently clustered based on the ligand RMSD. From the different clusters, 10 top-scoring disparate poses were selected manually (Figure S3), and each of these docked complexes were further simulated for 100 ns in MD to evaluate their overall stability within the active site of eLtaS. After the simulation, we analyzed the final snapshots from the simulations depicting the eLtaS active site and the positions of **1771** (Figure S4). Visual inspection of the snapshots revealed that poses 1, 2, and 9 had drifted away from the active site during the course of simulation. Hence, the simulations of these systems were discontinued at 70 ns. Time evolution of the **1771** RMSD also reflected the displacement of other poses from their original docked positions in the active site (Figure 1c). Out of the different poses simulated, only poses 4 and 10 showed a minimal deviation (<4 Å) from the initial docked conformations and stabilized during the simulation time.

Concurrent with our computational studies, we also expressed three eLtaS proteins in which the active site residues His416, His347, and Arg356 had been mutated to alanine, respectively (Figures S5–S9). These residues were chosen because our ensemble docking study suggested that the ester oxygen atom of **1771** interacts with His416, whereas the oxadiazole moiety of the ligand interacts with His347 and Arg356. Our isothermal titration calorimetry (ITC) results indicated that the His416Ala and Arg356Ala eLtaS variants exhibited a slight increase in binding affinities. However, mutating His347 to alanine abolished **1771** binding to eLtaS (Figure S9).

To evaluate the compatibility of the outcome of our mutagenesis studies with any of the simulated poses from MD, we plotted the minimum distance between His347 and Arg356 with the oxadiazole moiety of each simulated **1771** pose. Previous work by Richter et al. showed that the oxadiazole moiety is crucial for the biological activity of LtaS inhibitors.^30^ Out of the 10 poses simulated, only pose 4 maintained between the hydrogen-bonding distance of about 3 Å with His347 and Arg356, whereas all other poses failed to retain potential contact with these two residues (Figure S10). On the same lines, we also measured the minimum distance between the active site Mn^2+^ and the electronegative atoms of **1771** (Figure S11). Recent structural studies have shown that the Mn^2+^ coordinates with the phosphate head of GroP in the eLtaS active site.^40^ On this basis, we anticipated that an interaction with this metal ion could be crucial for **1771** to occupy the eLtaS active site. In this regard, the minimum distance between the Mn^2+^ ion and **1771** also went in accordance with the observation from other interaction analyses. Only pose 4 maintained a coordination distance of around 3 Å with Mn^2+^; all other poses were further from Mn^2+^ (more than 4 Å). Considering all these factors, we regarded pose 4 to be the most plausible representation of **1771** bound to the active site of eLtaS.

Previous in vitro kinetic studies using fluorescent-labeled lipids reveal that eLtaS cleaves the GroP head group of the PG lipid substrate to form the LTA backbone.^42^ Furthermore, based on the crystal structures of eLtaS from their work, Lu et al. have proposed a hydrolysis mechanism, whereby the GroP head group is coordinated to the Mn^2+^ and adopts a geometry that favors nucleophilic attack by the deprotonated Thr300.^40^ In the present work, our computational modeling reveals that the binding of **1771** apparently mimics the interaction of GroP with eLtaS. Similarities in the binding pattern of **1771** and GroP at the eLtaS active site suggest that **1771** could potentially function as a competitive inhibitor (Figure S12). In both instances, the two ligands can form hydrogen bonds with residues His347, Arg356, and His416. Additionally, the amide oxygen of **1771** and the phosphate oxygen of GroP are coordinated with the Mn^2+^. Notably, the Mn^2+^ remains coordinated by residues Glu255, Thr300, Asp475, and His476 in both cases. Meanwhile, the naphthofuran moiety of **1771** forms a π-stacking interaction with His476 and partially occupies the second GroP-binding site,^41^ which has been suggested to harbor the growing LTA polymer. This interaction could be crucial as Richter et al. showed that analogues of **1771** devoid of the naphthofuran ring exhibited weaker inhibitory activity.^30^ We also noted that the phenyl ring of **1771** is inserted deeply in a subpocket that is absent in the apo crystal structure (Figure 1d). This subpocket opens up as a result of a side chain rotation of His253 (Figure S13). This residue subsequently forms a π-stacking interaction with the phenyl ring of **1771**. Taken together, our observations suggest that **1771** mimics the interaction of GroP and prevents the latter from binding to the active site of eLtaS.

### Structure-Guided Optimization of **1771**

**Figure 2:**
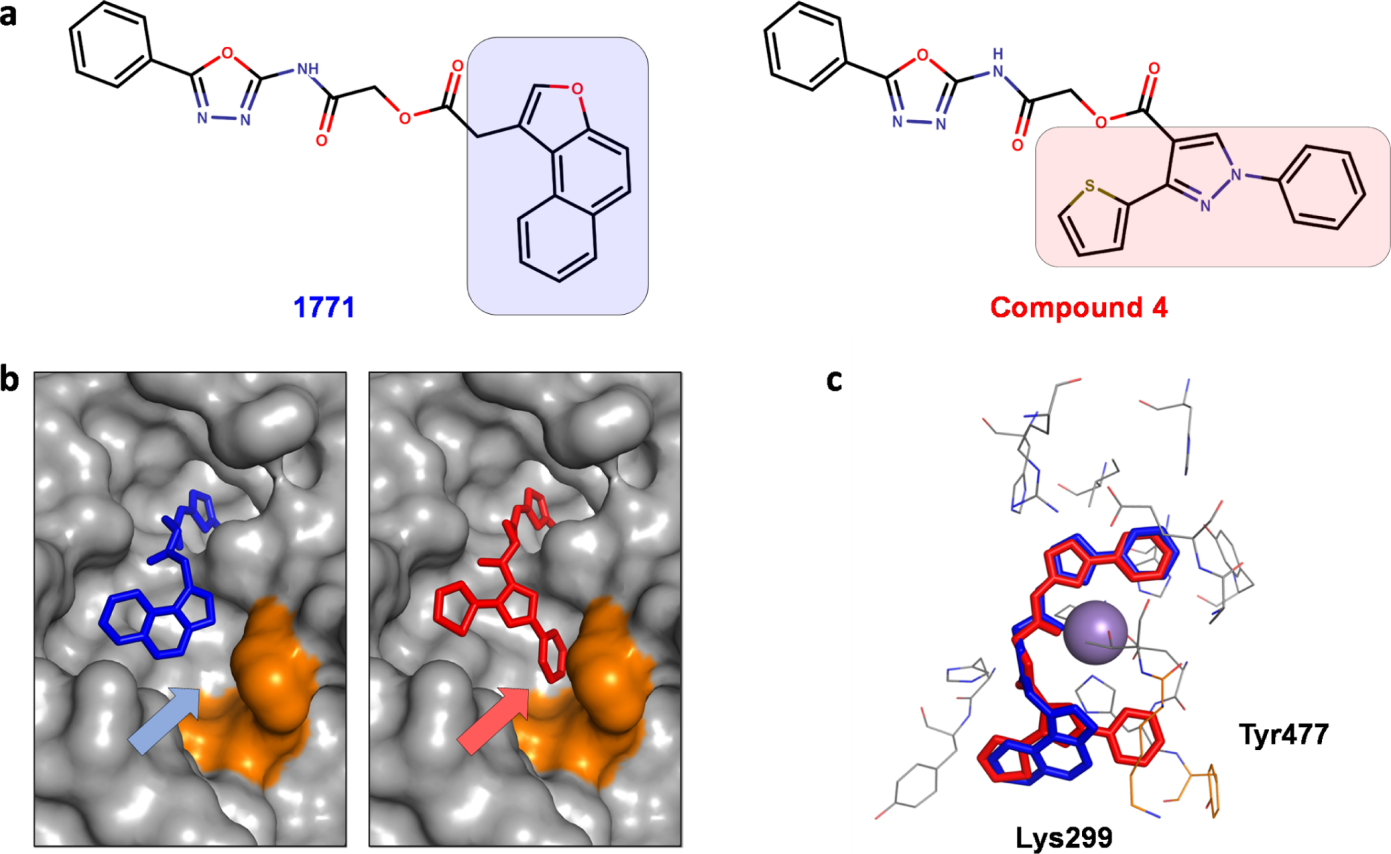
Structure-guided optimization of **1771**. (a) Chemical structure of **1771** and compound **4**. (b,c) Predicted binding pose of **1771** (blue stick) and compound **4** (red stick) in the eLtaS active site (shown in the gray surface and line representations). The residues Lys299 and Tyr477 are colored in orange.

Having established a working model of a **1771**-bound eLtaS, we attempted to identify analogues of **1771** with potentially improved antimicrobial potency. Visual inspection of the ligand-binding site revealed an unexploited binding cavity flanked by residues Lys299 and Tyr477 (Figure 2). These two residues are conserved among eLtaS-type enzymes and have been implicated in stabilizing the growing LTA chain in the pocket.^41^ We speculated that the inhibitor-binding affinity could be improved by modifying the naphthofuran ring to allow π-cation interaction with Lys299 or π-stacking interaction with Tyr477. For this purpose, we used ensemble docking to screen a customized virtual compound library containing various analogues of **1771**. We then visually inspected the top-scoring analogues for interaction with either Lys299 or Tyr477, and this led to a selection of 42 compounds for experimental testing. Of these analogues, seven compounds inhibited the growth of *S. aureus* with an IC_50_ ≤ 15 μM, but only compounds **3**, **4**, and **6** were able to reduce LTA production (Table 1 and Figure S14). To further compare the binding pose of compounds **3**, **4**, and **6** with **1771**, we conducted a small-scale molecular docking study of these compounds on eLtaS. Our docking results showed that neither the 4-methyl-1-,3-thiazole substituent of compound **3** nor the 3-methylbenzyl substituent of compound **6** were able to position themselves in the binding cavity lined by Tyr477 and Lys299. One plausible explanation is that the binding cavity could not accommodate the length of the substituents of compounds **3** and **6** (Figure S16).

**Table 1:** Antimicrobial IC_50_ of eLtaS Inhibitor Candidates^a^

compound	IC_50_ (μM)	compound	IC_50_ (μM)
**1**	15.31 ± 2.09	**5**	17.47 ± 1.08
**2**	12.34 ± 0.38	**6**	11.08 ± 0.49
**3**	13.40 ± 0.35	**7**	12.39 ± 1.90
**4**	4.06 ± 0.40	**1771**	14.90 ± 1.59

a The antimicrobial half maximal inhibitory concentration, IC_50_, values of the candidate compounds for eLtaS inhibition are shown. These compounds were selected on the basis that their antimicrobial potencies are similar or better than **1771**. The data represent the mean ± s.e.m. of *n* = 3 independent experiments, each performed in duplicate.

**Figure 3:**
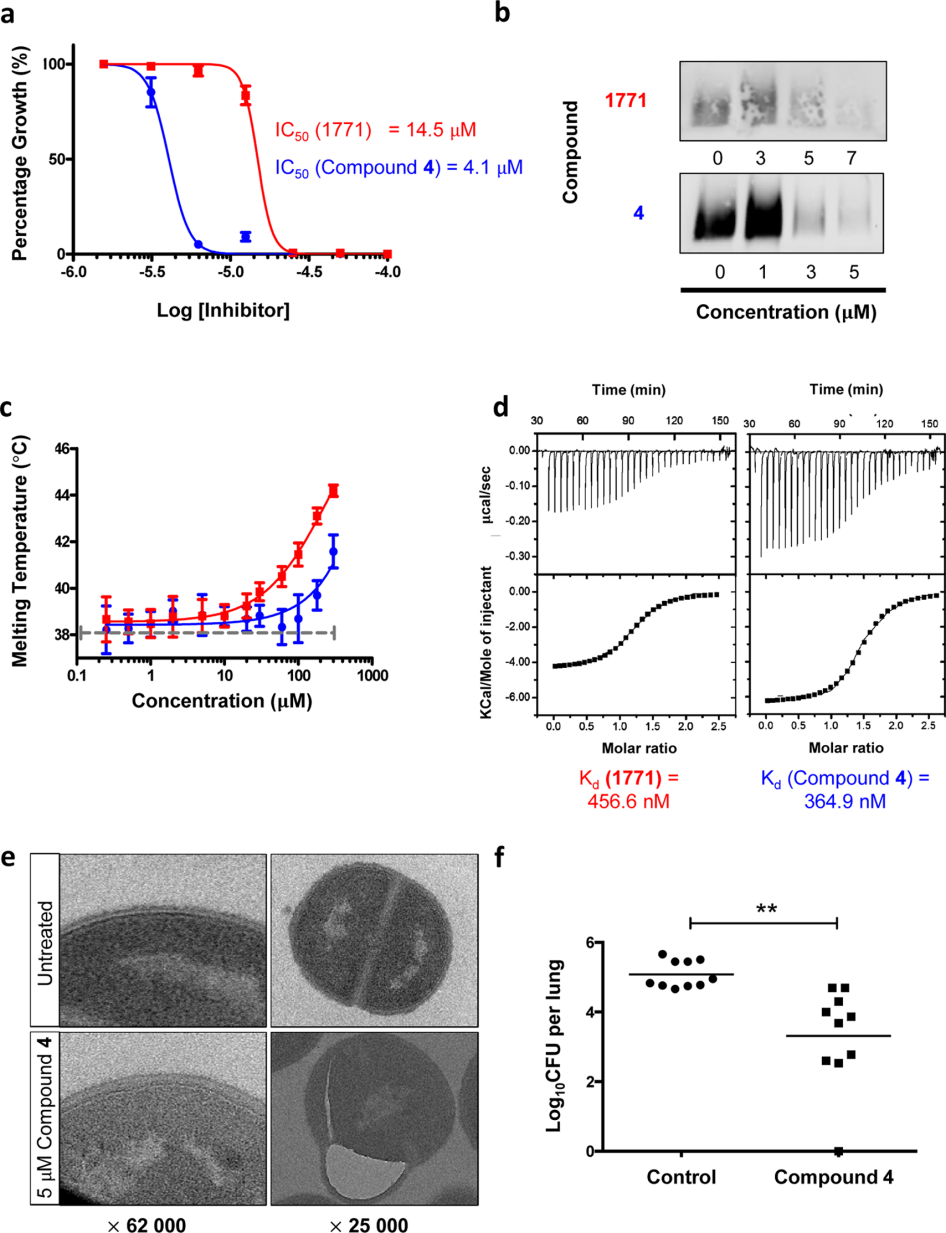
Biological activity and characterization of compound **4**. (a) Dose–response curves show the effect of **1771** (blue line) and compound **4** (red line) on *S. aureus* growth. (b) Immunoblotting of LTA in *S. aureus* treated with **1771** and compound **4** added at the indicated concentrations. The data shown are representative of at least three independent experiments. (c) Thermal stabilization of eLtaS in the presence of **1771** (blue line) and compound **4** (red line) across different concentrations as assessed by DSF. (d) ITC analysis of **1771** and compound **4** binding to wild-type eLtaS. Top panel of each thermogram depicts the raw calorimetric titration profile. Bottom panel shows the fitting of the experimental heat of binding to the model equations to derive the thermodynamic signatures. (e) TEM ultrastructure analysis of *S. aureus* without or with 5 μM compound **4**. (f) log_10_ CFU per lung of mice intranasally infected with *S. aureus* in the presence and absence of compound **4** treatment administered intranasally. Ten mice per group were used. **P-value < 0.01 when analyzed using a two-tailed Student *t*-test. Data in (a,c) represented as mean ± s.e.m. of *n* = 3 independent experiments, each performed in triplicate.

Due to its 4-fold improvement in its antimicrobial potency (Figure 3a), we decided to characterize compound **4** further. In this regard, Western blot analysis using *S. aureus* cell extracts showed that compound **4** was able to decrease LTA production in a dose-dependent manner (Figure 3b). Additionally, we also used differential scanning fluorimetry (DSF) to study the direct binding of compound **4** to eLtaS. Our DSF results indicated that compound **4** shifted the melting temperature of eLtaS more so than **1771** across the range of concentrations tested (Figure 3c). This suggests that compound **4** might be a stronger eLtaS binder than **1771**. This is based on the notion that the protein stabilizing effect of a compound is proportional to its affinity.^43,44^ These findings were supported by ITC experiments, which revealed that the binding affinity of compound **4** (*K*_d_ = 364.9 ± 6.8 nM) for eLtas was better than that of **1771** (*K*_d_ = 456.6 ± 7.1 nM; Figure 3d). Notably, the binding enthalpy of compound **4** was more exothermic than that of **1771**, which indicated that compound **4** forms additional interactions with eLtaS. Our docking studies suggest that these additional interactions arose from insertion of the phenyl moiety of compound **4** in the previously unexploited binding cavity.

Finally, thin-section transmission electron microscopy (TEM) showed that *S. aureus* treated with compound **4** displayed similar aberrant ultrastructures as LTA-deficient strains, including altered cell walls and erroneous placement of septa^39^ (Figure 3e). These data suggest that compound **4** induces *S. aureus* cell-wall stress and prevents proper cell division by inhibiting LTA production.

To investigate whether compound **4** could exert its effect in vivo, we first measured its cytotoxicity against HEK293 cells using a lactate dehydrogenase (LDH) release assay (Figure S17). Once we confirmed that compound **4** was non-cytotoxic, it was tested in a non-lethal *S. aureus* lung infection model. With a single dose, compound **4** led to a 2-log reduction in recoverable colonies from the lungs (Figure 3f). However, we were not able to compare the in vivo activity of compound **4** with **1771** because the mice did not tolerate the latter well in our hands. Taken together, these results suggest that compound **4** is an improved inhibitor of eLtaS that could decrease the number of bacterial titers in the lungs of mice infected with *S. aureus*.

### Identifying New Chemotypes as eLtaS Inhibitors

**Figure 4:**
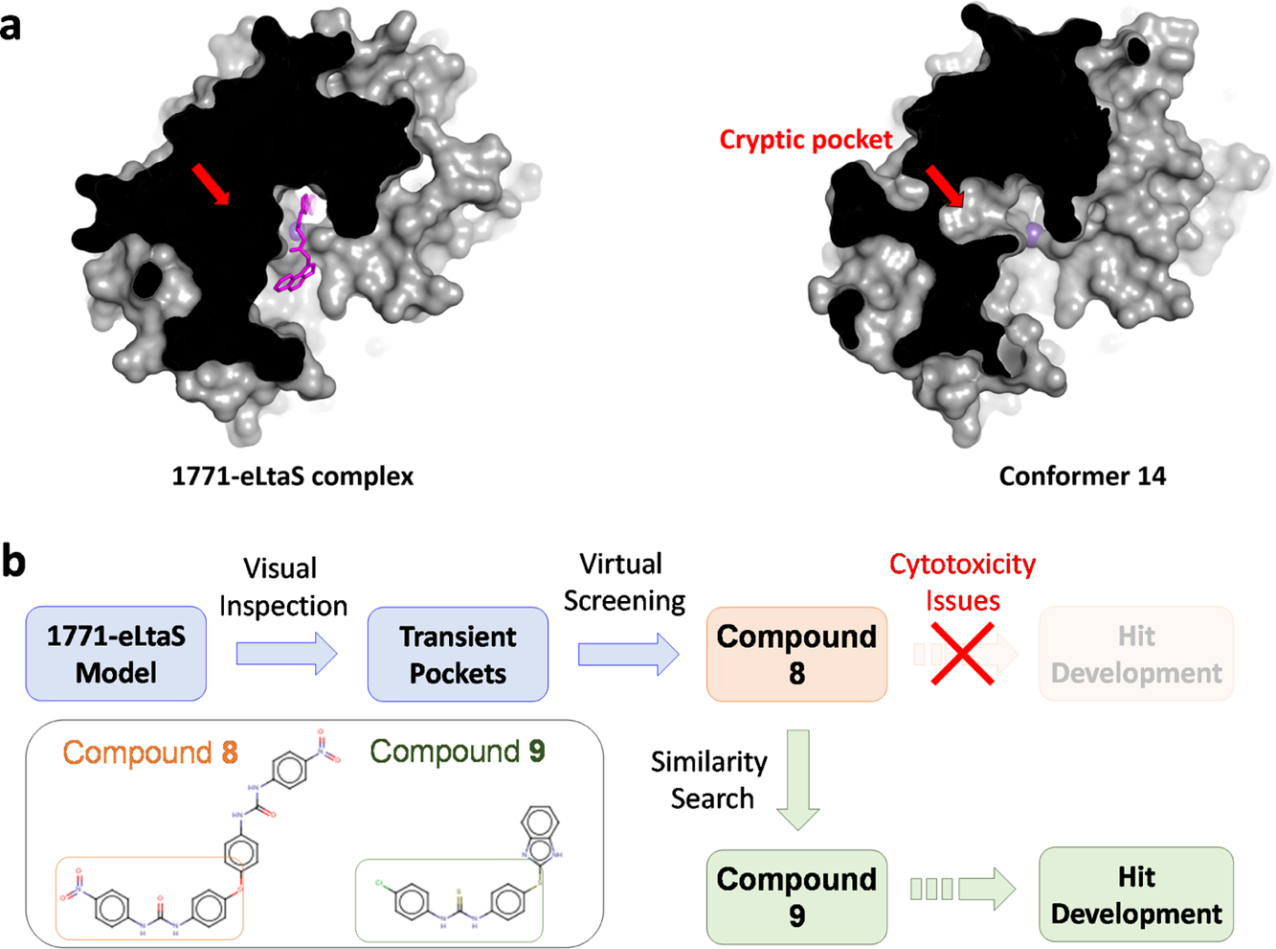
Targeting transiently open cryptic pockets of eLtaS. (a) Cryptic pocket that was absent in the **1771**-bound eLtaS model was observed in one of the conformers. (b) Schematic representation of the workflow used to identify new eLtaS chemotypes leading up to the discovery of compound **9**.

Our MD simulations indicated that the active site of eLtaS is highly flexible with the pocket volume fluctuating between 350 and 1600 Å^3^ over the 100 ns MD duration (Figure S18). Due to the dynamic nature of the active site, we identified several transiently open cryptic pockets that could be exploited to identify new chemotypes against eLtaS (Figure 4a). To assess the viability of this approach (Figure 4b), we conducted a small-scale VS of the NCI-Diversity Set V compound library by using ensemble docking. Although limited in size to only 1500 compounds, we chose this particular virtual library because of its wide chemotypic coverage over the compound chemical space.

From this feasibility study, we discovered compound **8**, a compound that bore a novel inhibitor scaffold. Western blot analysis indicated that the LTA extracted from *S. aureus* treated with compound **8** showed decreased electrophoretic mobility and higher heterogeneity. This suggests that compound **8** interferes with LTA production by a mechanism of action different from compound **4**. It is possible that the mechanism of action of compound **8** led to the production of LTA chains with increased length. Although compound **8** did not decrease LTA production, it was able to inhibit *S. aureus* growth (IC_50_ = 29.37 μM). We also noted from the sodium dodecyl-sulfate polyacrylamide gel electrophoresis (SDS-PAGE) analysis of the lysate that compound **8** upregulated a 24 kDa protein that was later identified by liquid chromatography/tandem mass spectrometry (LC/MS-MS) as IsaA, a peptidoglycan hydrolase (Figures S19 and S20).^45^ However, compound **8** was not a tractable hit because it did not have drug-like characteristics (MW > 400; clog *P* > 4)^46^ and was cytotoxic to mammalian HEK293 cells (Figure S17). Nevertheless, we reasoned that the chemical scaffold of compound **8** could be a starting point for finding other hits.

**Figure 5:**
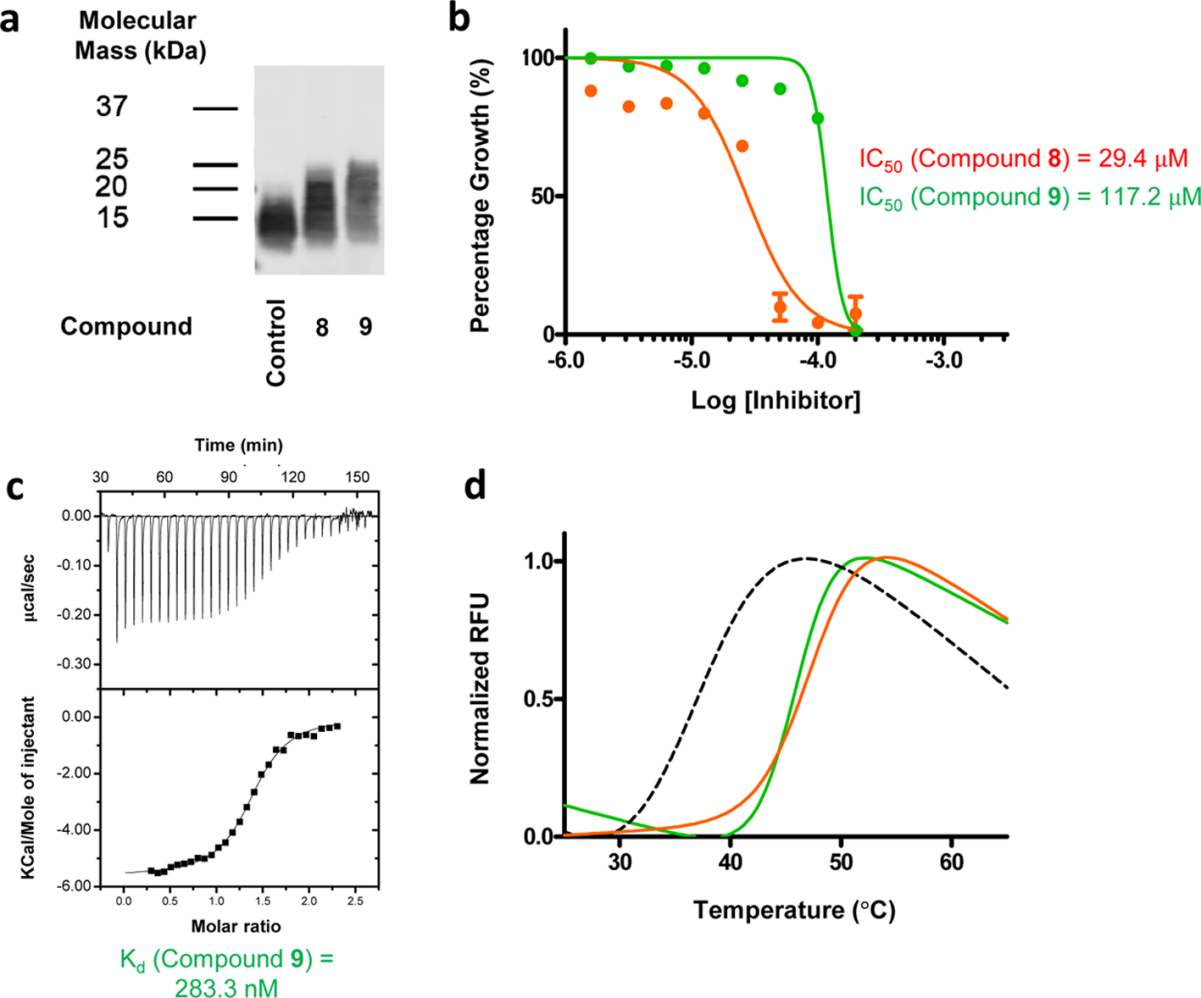
Biological and biophysical activity of compound **9**. (a) Immunoblotting of LTA in *S. aureus* treated with 100 μM of compound **8** or compound **9**. (b) Dose–response curves show the inhibitory effects of compound **8** (orange line) and compound **9** (green line) on *S. aureus* growth. Data represent mean ± s.e.m. of *n* = 3 independent experiments, each performed in duplicate. (c) ITC analysis of compound **9** binding to wild-type eLtaS. Top panel of each thermogram depicts the raw calorimetric titration profile. Bottom panel shows the fitting of the experimental heat of binding to the model equations to derive the thermodynamic signatures. (d) Thermal denaturation (*T*_m_) measurements of eLtaS in the presence of 100 μM of compound **8** (green line) or compound **9** (orange line). Thermal denaturation curve of the wild-type eLtaS is shown as reference (dotted gray line). The data shown in (a) and (d) are representative of at least three independent experiments.

Structurally, compound **8** is comprised of two chemically identical moieties (Figure 4b inset). We used one part of compound **8** as the starting “bait” to screen for analogues that are both non-cytotoxic and could retain similar bioactivity against eLtaS. For this purpose, we used ligand-based VS tools such as OpenEye’s ROCS and EON^47^ to screen the Enamine Advanced library for hits that exhibit similar pharmacophoric features as compound **8**. After visual inspection of the hits, we selected 23 compounds and tested them for bioactivity. From the purchased compounds, we discovered that compound **9** exhibited the same apparent mechanism of action as compound **8**. The LTA extracted from *S. aureus* cultures treated with compound **9** exhibited a similar “smearing effect” when analyzed by western blot (Figure 5a). We also confirmed that compound **9** was not cytotoxic when tested on HEK293 cells (Figure S17). Although compound **9** showed a 4-fold decrease in antimicrobial potency compared with compound **8** (IC_50_ = 117.24 μM; Figure 5b), its scaffold is more amenable for hit-to-lead optimization. However, and unlike compound **8**, we found that compound **9** binds directly to eLtaS using ITC and DSF (Figure 5c,d). Taken together, these data suggest that compound **9** interferes with the LTA synthesis process and inhibits bacterial growth.

**Figure 6:**
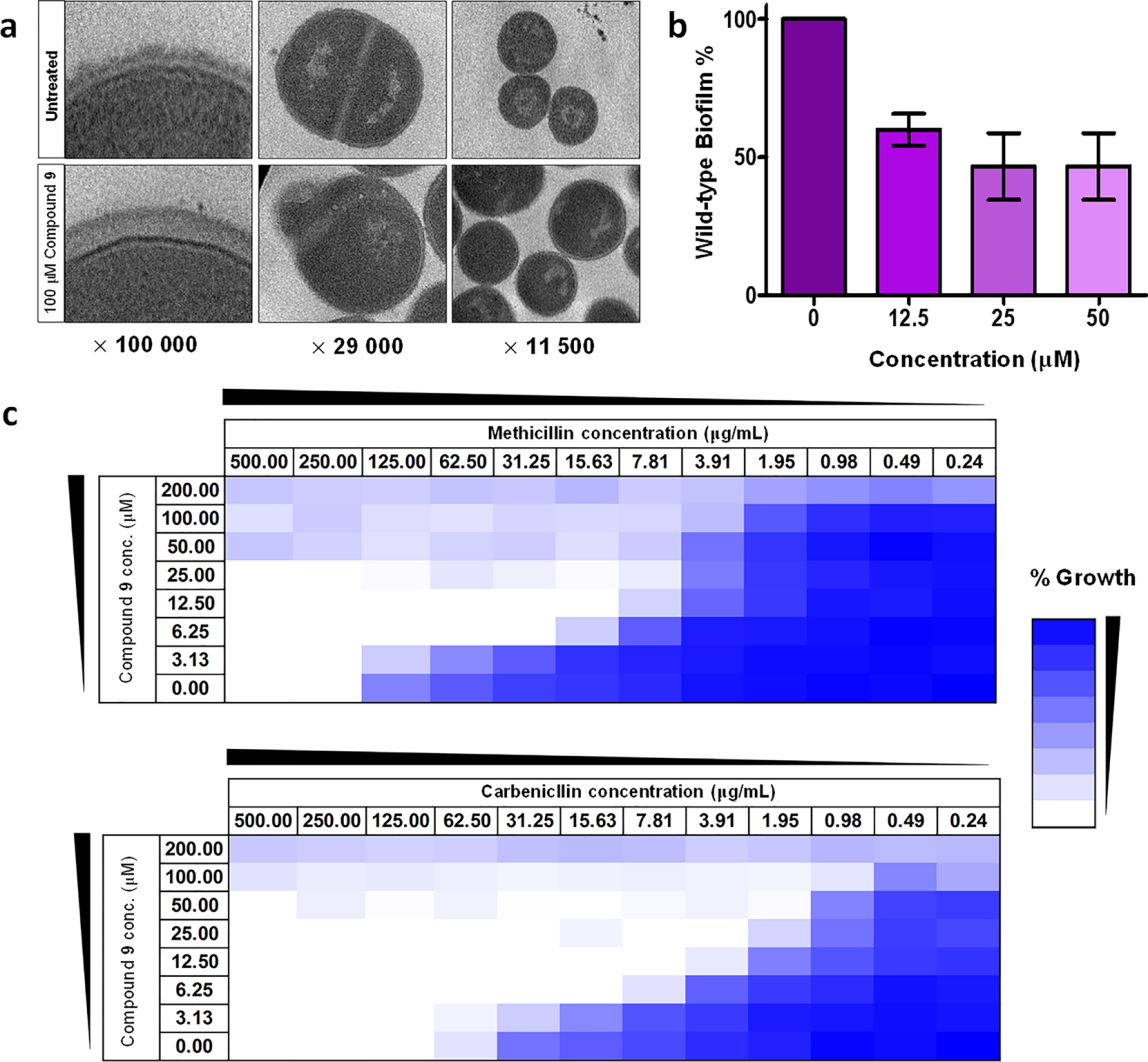
Characterization of compound **9**. (a) Ultrastructure of *S. aureus* treated without or with 100 μM compound **9** was viewed under a TEM microscope. (b) Synergistic effect of β-lactams (methicillin or carbenicillin) and compound **9** against MRSA is assessed using a microdilution checkerboard analysis. Percentage growth is shown as a heat plot. Four independent experiments were replicated. (c) Effects of compound **9** on *S. aureus* biofilm formation when tested at the indicated concentrations. Data represented as mean ± s.e.m. of *n* = 3 independent experiments, each performed with four technical replicates.

TEM studies revealed that compound **9** treatment resulted in swollen *S. aureus* cells with a thickened peptidoglycan layer and cell division defects (Figure 6a). Also, *S. aureus* treated with compound **9** showed a 50% reduction in biofilm attachment (Figure 6b). This was in agreement with previous work showing that a mutant stain of *S. aureus* with decreased LTA content exhibits decreased biofilm formation activity.^48^ More importantly, in an antimicrobial synergy study, we observed that compound **9** could potentiate β-lactam activity against MRSA. At a subinhibitory concentration of 12.5 μM, compound **9** reduced the minimum inhibitory concentration of methicillin and carbenicillin against MRSA (strain COL) by 16- and 32-fold, respectively (Figure 6c). In addition, the MRSA strain treated with compound **9** had a 4-fold increase in sensitivity toward polymyxin B (Figure S21). In view of these findings, we suggest that further optimization of compound **9** could lead to derivatives that are clinically relevant in fighting against MRSA infections.

## Discussion

In our present study, we modeled an inhibitor-bound eLtaS structure by using a systematic computational methodology to direct our drug design. We coupled “blind” and ensemble docking methods together with MD simulations to model a **1771**-bound eLtaS complex. We then validated our model by showing that mutations of residues in the binding site perturbed the binding thermodynamics of **1771**. Using structural insights derived from the model, we then conducted a VS campaign for lead-optimization and hit-discovery. From these efforts, we discovered two new inhibitors: compound **4**, which is an improved inhibitor of eLtaS, and compound **9**, which is a new chemotype that exhibited a different mode of action compared with compound **4**. We also provided evidence that targeting eLtaS could decrease biofilm formation and resensitize MRSA to β-lactam action. Taken together, our work serves to provide a computational platform to rationally design inhibitor against LtaS.

A key challenge of applying structure-based VS to promising antimicrobial targets is the lack of any inhibitor-bound crystal structure to guide the molecular docking process. Docking studies that rely purely on apo crystal structures are often a cause for concern because the ligand-binding pockets may have collapsed or been occluded by side chain movements. Our current study with eLtaS provides a case in point. Structurally, the active site of eLtaS is surrounded by several flexible protein loops which may reshape the active site so that eLtaS can fulfil its numerous functions such as recognizing the phospholipid substrate, stabilizing the enzymatic transition state and identifying the LTA glycolipid anchor for GroP attachment. In our MD simulation, we observed a conformational change by His253 that opened up a subpocket for **1771** to bind into. This subpocket is absent in the crystal structure of apo eLtaS. Hence, rigid-receptor docking onto just the crystal structure could have derailed our VS campaign. Therefore, in a broader context, our work underlines the importance of considering protein flexibility in modeling inhibitor–protein complexes and for VS.

If modeled correctly, inhibitor-bound protein complexes from computational predictions could be helpful in optimizing existing inhibitors or in discovering new ligands. This study provided two examples of how structural insights derived from our **1771**-bound eLtaS model led to the discovery of two inhibitors. The first example was our lead-optimization process, where we noted that **1771** did not interact with the subpocket lined by Lys299 and Tyr477. Hence, our VS focused on discovering inhibitors that could form a π-stacking interaction with Tyr477. This process yielded compound **4**, which was able to outperform **1771** in our antimicrobial and biophysical assay. The second example was our attempt to identify novel chemotypes by targeting the transient pockets that appeared intermittently over the duration of the MD simulation. Studies on other protein systems have shown that targeting transient pockets could lead to new chemotypes.^49−51^ In our situation, this approach led to the discovery of compound **9**. The LTA of *S. aureus* treated with compound **9** exhibited lower electrophoretic mobility on the SDS-PAGE gel. This lower mobility was similarly observed in the LTA extracted from *S. aureusypfP* and *ltaA* mutant strains.^48^ Functionally, the glycosyltransferase YpfP synthesizes the glycolipid anchor Glc_2_-DAG for LTA attachment inside the bacterial cytoplasm. The anchor is subsequently flipped by LtaA to the exterior of the cell membrane.^52,53^ In both mutants, the LTA is incorrectly attached to DAG.^48,52^ On this basis, we hypothesize that compound **9** exerts its activity by interfering with LTA attachment onto the glycolipid anchor. This mechanism is different to that proposed for **1771** and compound **4**, which behaved as a competitive inhibitor of eLtaS. Our docking studies support a difference in the mode of action for these two inhibitors based on the different binding pose of **1771** and compound **9** (Figure S22). As compound **9** did not exhibit cytotoxicity against the HEK293 mammalian cells, the compound shows promise for further development as a β-lactam potentiator. As such, our future work will focus on derivatizing compound **9** to improve the potency and solubility of this inhibitor scaffold.

Finally, the two inhibitors from our work further reinforce the notion that targeting LtaS using small molecules is a viable strategy to combat *S. aureus* infections. Previous work using LtaS-knockouts or LtaS inhibitors such as **1771** has shown that depletion of LTA leads to deleterious effects in *S. aureus*.^30,52^ To our knowledge, our paper is the first to show that interfering with LTA synthesis can prevent bacterial growth, even if done so without full inhibition of LTA production. Recent studies have alluded to the fact that downregulating LtaS expression leads to resensitization of MRSA to β-lactam antibiotics.^54,55^ Because β-lactam antibiotics are still considered as one of the safest and most efficacious antibiotics available, adjuvants such as β-lactamase inhibitors have been developed to potentiate β-lactam against antibiotic-resistant bacteria.^56,57^ We found that small molecules that interfere with LTA synthesis, such as compound **9**, could be used for that purpose too. Additionally, LTA is implicated in biofilm formation by Gram-positive bacteria.^48,58^ Biofilms are sessile aggregates of bacteria that grow on both biotic and abiotic surfaces. They are often associated with antimicrobial resistance and catheter-related infections in hospitals.^59−61^ In this regard, our biofilm assay data suggest that compound **9** could also be used to inhibit biofilm formation.

To conclude, the epidemic of antibiotic-resistant infections has forced researchers to look for new antimicrobial targets. To this end, targeting LtaS is a proven proposition for future development of antimicrobial drugs and our work provides the foundation for rationally design inhibitors against this target. In a broader perspective, our work also further reinforces the notion that conformational flexibility is an important consideration when conducting a VS campaign. When combined with proper knowledge of the protein target, and perhaps with a bit of serendipity, VS can be a very powerful repertoire in the pipeline of drug discovery.

## Methods

### MD Simulations

An all-atom MD simulation of the wild-type extracellular LtaS domain (eLtaS) was performed using the crystal structure 2W5Q.^40^ For the preparation of the eLtaS model, the H++ web server^62^ was used to add the hydrogen atoms on each protein residues with the protonation state at pH 6.5. In the 2W5Q structure, the Mn^2+^ in the eLtaS active site is co-ordinated with the residues Glu255, Asp475, His476, and Thr300. Thr300 was kept deprotonated because it was predicted to be bound to the Mn^2+^ ion with the hydroxyl group in that form.^40^ The Mn^2+^ ion was replaced with the octahedral dummy atom mode of manganese ions described by Duarte et al.^63^ Energy minimization in vacuum was carried out with the sander module of the Amber 14.0 simulation package.^64^ The minimized protein was then solvated in a cubic periodic box of TIP3P water model with water molecules extending 14 Å outside the protein complex on all sides. Overall, the simulation box contained 16413 TIP3P water molecules, and the charge neutrality was maintained by adding 11 Cl^–^ ions.

For all the subsequent simulations, the AMBER 14.0 simulation software package with the AMBER ff99SB force field was used. Additionally, the SHAKE algorithm was used to constrain all bonds involving the hydrogen atoms for the simulation.^64^ Prior to the simulation, the temperature was increased to 300 K in the canonical ensemble, and the system was equilibrated for 10 ns in the NPR ensemble, with 2 fs simulation time step. During this period, the energy components and system density were seen to be converging (data not shown). Subsequently, the system was further simulated to generate 100 ns of production data. The long-range electrostatic interactions were calculated using particle mesh Ewald sum with a cutoff of 10 Å applied to Lennard-Jones interactions. The simulation trajectories were saved at an interval of 2 ps for further analyses. The overall motion of eLtaS over 100 ns was analyzed with PCA by using the CPPTRAJ module of AMBER 14.0.

An RMSD-based conformational clustering algorithm implemented in CPPTRAJ was also used to generate a reduced dataset of the 100 ns long simulation trajectories. Representative cluster centroids were used for ensemble-based docking and VS procedures. The visual analysis of protein structures were carried out using PyMOL and VMD.^63^

### Binding Site and Pose Prediction

Prior to docking, the structure of **1771** was drawn using MarvinSketch version 15.2.2. and energy-minimized using the AMBERff99 forcefield implemented in UCSF Chimera version 1.11.2. Meanwhile, the protein conformers were obtained directly from MD.

The “blind” docking protocol described by Hetényi and van der Spoel^65^ was used to determine the plausible binding site of compound **1771**. In this protocol, the ligand search space (144 Å × 156 Å × 163 Å) of AutoDock 4.2 was set to cover the entire eLtaS protein. The number of binding modes generated per run was set to “9” and the exhaustiveness of search was set at the default value of “8”. For each of the 20 conformers (PDB-ID 2W5Q and the nineteen MD-generated conformers), 100 docking runs were run using the docking settings above. Hence, 900 docked poses were generated for each conformer; however, only the top 100 poses for each of them were considered for clustering analysis.

Once the putative binding site of **1771** had been determined through blind docking, focused docking using the molecular docking GOLD suite version 5.3.0 (CCDC, Cambridge, UK) was used to predict the binding pose of **1771** at the binding site. For this purpose, the docking search space was set to cover all atoms within a 20 Å sphere centered on the Mn^2+^ ion. The GOLDScore fitness scoring function and the standard genetic algorithm sampling protocol were used for the docking runs. In total, 200 independent focused docking runs were conducted from GOLD and all of the top-ranked poses from each docking run were visually inspected and clustered into 10 bins. Subsequently, a representative pose from each bin was chosen and simulated for an additional 100 ns in MD using the aforementioned protocol to assess their stability in the binding site.

### VS Leading to Compound **4**

ROCS version 3.2.1.4 and EON version 2.2.0.5 (OpenEye Scientific Software, NM, USA) were used to screen a conformer library made from the Enamine Advanced library (containing ∼482,000 compounds) to produce a list of molecules that shared similarity with compound **1771** in 3D shape and surface electrostatics. These compounds were subsequently docked to the eLtaS using GOLD suite following the aforementioned protocol. The ligand-interaction profiles for the 100 top-scoring compounds were visually inspected to identify compounds that are predicted to have interactions with the subpocket lined by Lys299 and Tyr477. Finally, 42 compounds were selected by this method and purchased for experimental testing. The purity data of compounds **1771** and **4** were determined by the vendor Enamine Ltd using LC–MS (Figures S23 and S24). The compound catalog IDs for compounds **1771** and **4** are Z25275760 and Z18903036, respectively.

### VS Leading to Compound **9**

The Diversity Set V library (containing ∼1600 compounds) was docked using GOLD directly following the protocol above. The top 40 compounds ranked by GOLDScore were ordered from the National Cancer Institute as part of the service of the Developmental Therapeutic Program (DTP). Following the discovery of compound **8**, the enamine advanced conformer library was screened using ROCS and EON to search for analogues of compound **8**. The top 40 compounds (including compound **9**) ranked by EON were purchased for experimental testing. The purity data of compound **9** were determined by the vendor Enamine Ltd using LC–MS and NMR spectroscopy (Figure S25). The compound catalog ID for compound **9** is Z45900028.

### Antimicrobial and Potentiation Assay

All the compounds tested in this study were dissolved in dimethyl sulfoxide (DMSO) at an initial stock concentration of 20 mM and stored at −20 °C. These compounds were tested for their antimicrobial activity using the microdilution broth method^66^ following the protocol described by Richter et al.^30^ Briefly, overnight cultures of *S. aureus* (strain Newman) grown at 37 °C in cation-adjusted Mueller Hinton II (MH2) broth were adjusted to an OD_600nm_ of 0.6 before back-diluted with a factor of 1:2000 with the same broth. After that, 200 μL of the diluted bacterial culture were dispensed into each well of 96-well plates supplemented with the compounds to the desired concentrations. The plates were then sealed with a moisture membrane barrier and incubated statically for 20 h at 37 °C. After that, the OD_600nm_ values of the cultures in each well were measured using a CLARIOstar microplate reader (BMG Labtech). The procedures for the potentiation assays were by large the same as described above with some variations. In such assays, MRSA strain COL was grown in the 96-well plates supplemented with different concentrations of compound **9** with methicillin, carbenicillin, or polymyxin B in a checkerboard format. Their synergistic effects were quantified by OD_600nm_ measurements using a CLARIOstar microplate reader (BMG Labtech).

### Biofilm Assay

Biofilm assays were carried out on 96-well flat-bottom plates using the protocol described by Fedtke et al.^48^ Briefly, *S aureus* (strain Newman) was grown statically in 96-well plates at 37 °C in Tryptic soy broth supplemented with 0.5% glucose. Each well was additionally supplemented with either DMSO or compound **9** to concentrations of 50, 25, and 12.5 μM. After 20 h, the bacterial cultures from the plates were removed, and each well was rinsed with 150 μL of sterilized distilled water thrice. The plates were then incubated for 1 h at 60 °C. After that, the biofilm in each well was stained with 0.05% (w/v) of crystal violet solution using the protocol described by O’Toole.^67^ Finally, the stained biofilms were solubilized with 30% acetic acid and quantified at OD_600nm_ using the BMG Labtech CLARIOstar microplate reader.

### Western Blot

The procedures of LTA detection using Western Blot had previously been reported by Gründling et al.^52^ To determine the effect of the compounds on LTA production, 10 mL cultures of *S. aureus* (strain RN4220) in MH2 broth were incubated in an orbital shaker at 37 °C with either the compounds or DMSO. When the control cultures had reached an OD_600nm_ ∼ 1, the bacteria were pelleted and the OD_600nm_ of the cultures normalized by resuspension with the appropriate amount of MH2 broth. Subsequently, 1 mL from each cultures was mixed with 0.5 mL of 0.1 mm glass beads, and the bacteria were lyzed by vortexing at 4 °C for 45 min. After lysis, glass beads were removed by centrifugation at 200*g*, and the supernatants were transferred to a new tube. The membrane-associated LTA from each culture were pelleted by an additional centrifugation step of the supernatant at 16,000*g* for 15 min before resuspension in 30 μL of 2× SDS. The samples were boiled at 80 °C for 20 min, and the solubilized fractions were loaded onto 15% SDS-polyacrylamide gels prior to electrophoresis at 120 V for 1.5 h. The LTA on the gels were electrotransferred onto FL-immobilon PVDF membranes at 25 V for another 1.5 h. After the electrotransfer, the membranes were blocked with 5% skimmed milk for 1 h and incubated with the antimouse LTA antibody (clone G43J, Thermo Fisher Scientific; 1:1000 dilution) overnight at 4 °C. After washing, the membranes were incubated with antimouse IRDye 680RD (1:10,000 dilution) for 1 h, washed, and visualized using the Odyssey CLx Imaging System.

### Cell Viability Assay

The human embryonic kidney (HEK293) cells were maintained in Dulbecco’s modified Eagle medium (DMEM) supplemented with 10% fetal bovine serum and 100 units/mL of penicillin–streptomycin. For the cell viability assays, cells were seeded onto 96-well plates at a density of 4 × 10^4^ cells/well and grown at 37 °C at 5% CO_2_. After incubating the cells for 24 h, the culture media were replaced with fresh DMEM supplemented with 200 μM of the compounds **1771**, compound **4**, compound **8**, and compound **9** and incubated further for an additional 24 h. The cytotoxicity of these drugs was measured using the LDH Cytotoxicity Assay Kit according to the manufacturer’s instruction (Thermo Scientific). The absorbance at 490 nm was measured with a CLARIOstar microplate reader (BMG Labtech), and the reference wavelength was set at 630 nm.

### Thin-Section TEM

The TEM imaging protocol was adapted from Garufi et al.^68^*S. aureus* (strain Newman) was grown in 20 mL of MH2 broth with either DMSO, 5 μM of compound **4** or 100 μM of compound **9** at 37 °C. When the control culture had reached an OD_600nm_ ∼ 1, the cells were pelleted at 1520*g* and submitted to the Cambridge Advanced Imaging Centre for preimaging processing. Briefly, the cells were washed with water and fixed with 2% glutaraldehyde-4% paraformaldehyde in 0.1 M sodium cacodylate buffer (pH 7.2) for 2 h. After fixation, the cells were stained, dehydrated, and embedded in Spurr resin. The resin was cut into thin sections and viewed using a Tecnai G2 Transmission Electron Microscope at 200 kV with a bottom-mounted AMT CCD digital camera.

### Recombinant Protein Expression and Purification

The plasmid [Rosetta pProEX-eLtaS] from *E coli* strain ANG571 was extracted using the QIAprep Spin Miniprep Kit. Meanwhile, the genes encoding the mutant LtaS with mutations His416Ala, His347Ala, and Arg356Ala were extracted from the strains ANG1115, ANG1175, and ANG1178, respectively, using the QIAamp DNA Mini Kit. These genes were amplified using PCR, cut with NdeI/BamHI, and ligated into a pET19m transformed into *E. coli* Rosetta 2 (DE3) strain.

Each of the eLtaS proteins were purified from 6 L of *E. coli* cultures that were grown with aeration in LB medium supplemented with 200 μg/mL of carbenicillin and 34 μg/mL of chloramphenicol at 37 °C. When the cultures reached an OD_600nm_ of about 0.6, protein expression was induced with 1 mM IPTG final concentration at 21 °C overnight. Later, the bacteria were harvested by centrifugation at 11,899*g*, and the bacterial pellets were resuspended with lysis buffer (50 mM Trizma-HCl, pH 7.5, 300 mM NaCl, 5% glycerol, 10 mM imidazole, 5 mM β-mercaptoethanol). The bacterial pellets were lyzed by three passages through a high-pressure emulsifier, and the lysates were centrifuged at 47,596*g* for 30 min at 4 °C. The resulting supernatants containing the His-tagged eLtaS proteins were filtered with a 0.22 μm filter before being loaded onto a 5 mL Ni-NTA column using an ÄKTApure protein purification system. The bound proteins were subsequently washed with lysis buffer, eluted using the elution buffer (50 mM Trizma-HCl, pH 7.5, 300 mM NaCl, 5% glycerol, and 300 mM imidazole), and dialyzed twice using the dialysis buffer [25 mM Trizma-HCl, pH 7.5, 100 mM NaCl, 10% glycerol, 1 mM ethylenediaminetetraacetic acid (EDTA)]. After dialysis, protein purity was confirmed to be >90% using SDS-PAGE. Proteins were then concentrated, snap-frozen in liquid N_2_, and stored at −80 °C prior to usage. All four eLtaS proteins were subjected to circular dichroism analysis, and their exact masses were determined using MS.

### Differential Scanning Fluorimetry

DSF assays were performed on eLtaS using the CFX Connect RT-PCR system (Bio-rad) using the protocol described previously.^43^ Solutions containing 5 μM eLtaS protein in DSF buffer (25 mM Trizma-HCl, pH 7.5, 100 mM NaCl, 10% glycerol, 1 mM EDTA, 20× SYPRO Orange) with compounds at the appropriate concentrations were dispensed onto 96-well PCR plates in triplicate. The plates were sealed with Microseal “B” PCR Plate Sealing Film (Bio-rad) prior to the DSF run. The emitted fluorescence at 568 nm was measured from 25 to 65 °C at a step ramp rate of 0.5 °C every 30 s. Prism GraphPad v.5 was used for curve fitting, and the melting temperature *T*_m_ for each curves was derived from the Boltzmann equation.

### Isothermal Titration Calorimetry Assay

The thawed eLtaS proteins were dialyzed using D-Tube Dialyzer Mini (EMD Millipore) in 20 mM Trizma-HCl pH 7.5, 100 mM NaCl with Chelex 100 (Sigma-Aldrich) for 1.5 h twice on the same day prior to the ITC experiments. After dialysis, the eLtaS proteins were centrifuged at 20,000*g* at 10 °C for 10 min. The supernatants were transferred to new microcentrifuge tubes and diluted to 100 μM using the same buffer as above with an addition of 0.1% (v/v) DMSO. Meanwhile, the eLtaS inhibitors were diluted to 10 μM using the same buffer and maintaining 0.1% (v/v) DMSO. The eLtaS proteins were titrated into the main cell containing the inhibitors using a VP-ITC at 25 °C. The injection parameters were set as follows: a reference power of 15 μcal/s, an initial injection of 3 μL over a duration of 3.6 s, and then subsequent injections of 10 μL over a duration of 12 s. All injections were spaced with 240 s with a filter period of 2 s. Data were first analyzed in NITPIC^69^ for baseline calculations and then fit for thermodynamic parameters using the OneSite model in Origin 7.0 software.

### Lung Infection Mouse Model

All in vivo experiments were done at the University of Liverpool under the UK Animals (Scientific Procedures) Act 1986 guidelines. The protocols were approved by the UK Home Office and by the University of Liverpool Animal Welfare and Ethics Committee. Six to eight week old BALB/c female mice (Charles River, UK) were used in the study after being left to acclimatize for a minimum of 7 days prior to experimentation. Mice were intranasally infected with 5 × 10^7^ CFU of the *S. aureus* (strain Newman) in 50 μL of sterile phosphate-buffered saline (PBS). The bacterial dose was prepared by incubating 50 μL of an overnight culture in brain heart infusion (BHI) broth (Oxoid, Thermo Fisher Scientific, UK) into 5 mL of fresh BHI. Once the culture reached an OD_600_ = 2–3, the bacterial numbers were adjusted to the desired dose using sterile PBS. Three hours postinfection, mice were intranasally treated with either 50 μL of sterile PBS (control) or with 300 μg of compounds **1771** and compound **4** dissolved in 50 μL of sterile PBS. The final DMSO concentration was 5%, and the PBS control also contained 5% DMSO. Mice were culled 24 h postinfection; lungs were collected, homogenized, and serially diluted to determine CFU counts.^70^ Significant differences were determined using the unpaired two-tailed Student *t*-test (*p* = 0.0013).

## Data and Software Availability

Blind docking was conducted using AutoDock 4.2.0 on the PyRx (version 0.8) VS platform. Focused docking was conducted using the Genetic Optimization for Ligand Docking (GOLD; version 5.3.0) using the free academic license courtesy of the Cambridge Crystallographic Data Centre (CCDC). All MD simulations were conducted using the Assisted Model Building with Energy Refinement (AMBER; version 14.0) using a purchased academic license.

## Supporting Information Available

Selection of eLtaS conformations for ensemble docking; docking poses of compound **1771** before and after MD simulations; structural integrity data of eLtaS mutants; alanine-mutagenesis studies of compound **1771** binding to eLtaS mutants; interactions and probable binding pose of compound **1771** in the eLtaS active site; experimental and molecular docking data of shortlisted eLtaS inhibitor candidates; dynamic nature of the eLtaS active site; identification of upregulated lytic transglycosylase in *S. aureus* treated with compound **8**; antibiotics potentiation data of compound **9** against MRSA; predicted binding pose of compound **9** in eLtaS; purity data of compounds **4**, **1771**, and **9**; and identification of potential eLtaS inhibitor-binding site using a blind docking protocol (PDF)
