# Supplementary material for: Structure-Based Discovery of Lipoteichoic Acid Synthase Inhibitors
Source: J Chem Inf Model. 2022 May 9;62(10):2586–99. doi: 10.1021/acs.jcim.2c00300 (PMC9131456; doi:10.1021/acs.jcim.2c00300)
Supplement: Supplementary file 1 — ci2c00300_si_001.pdf [file ci2c00300_si_001.pdf]

SUPPORTING INFORMATION

Structure-based discovery of Lipoteichoic acid Synthase inhibitors

Xavier Chee Wezen[1,*], Aneesh Chandran[2,*], Rohan Sakariah Eapen[1,*], Elaine Waters[3], Laura Bricio Moreno[3], Tosi Tommaso[4], Stephen Dolan[5], Charlotte Millership[4], Aras Kadioglu[3], Angelika Gründling[4], Laura Itzhaki[1], Martin Welch[5,**] & Taufiq Rahman[1,**]

[1]Science Program, School of Chemical Engineering and Science, Faculty of Engineering, Computing and Science, Swinburne University of Technology Sarawak, Kuching 93350, Malaysia
[2]Molecular Biophysics Unit, Indian Institute of Science, Bangalore 560012, India.
[3]Department of Clinical Infection Microbiology and Immunology, Institute of Infection and Global Health, University of Liverpool, Liverpool L69 7BE, UK.
[4]MRC Centre for Molecular Bacteriology and Infection, Imperial College London, London SW7 2AZ, UK.
[5]Department of Biochemistry, University of Cambridge, Cambridge CB2 1QW, UK.

*These authors contributed equally to this work.
**These authors jointly supervised this work. Correspondence and requests for materials should be addressed to M.W. (email: mw240@cam.ac.uk) or to T.R. (email: mtur2@cam.ac.uk)

SUPPORTING INFORMATION IN THIS DOCUMENT:

Supporting Figures: S1-S25

Supporting Tables: ST1

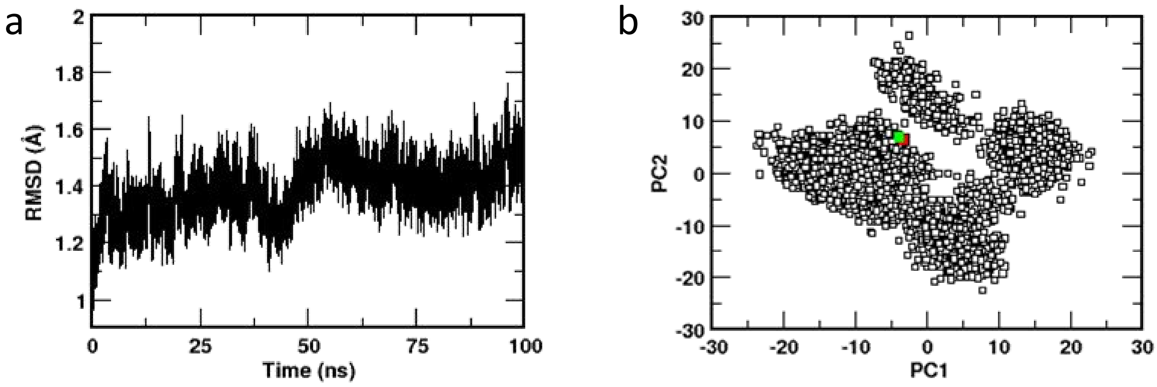

Figure S1. Conformational landscape of the simulated eLtaS. (**a**) The RMSD plotted represents the protein backbone movement of the simulated eLtaS across the simulation timeframe. The RMSD is calculated with respect to the conformation of the original crystal structure (PDB-ID 2W5Q). (**b**) Two-dimensional projection of the simulated structures of eLtaS on a plane. The plane is constituted by the most significant principal components (PC1 and PC2) obtained from the principal component analysis of all the sampled eLtaS conformations (black squares). Crystallographic structures of the apo (red; PDB-ID 2W5Q) and GroP-bound (green; PDB ID 2W5T) eLtaS are shown as squares on the 2D-plane.

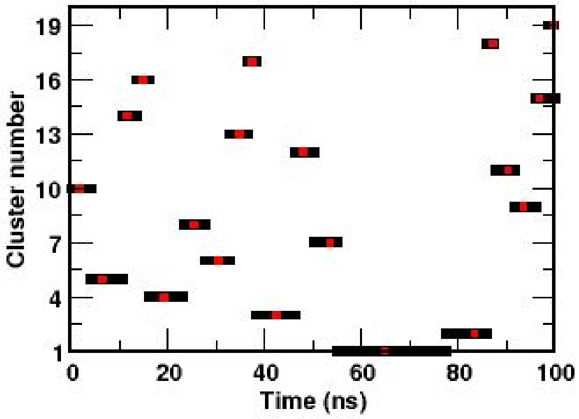

Figure S2. Selection of the nineteen centroids to form the eLtaS ensemble. Figure shows the RMSD-based clustering of all the eLtaS conformations sampled by MD. Clusters are arranged in ascending order i.e. cluster 1 is the most populated conformation and cluster 19 is the least populated conformation from the simulated trajectories. The representative structures for each conformation cluster (centroids) are shown in red.

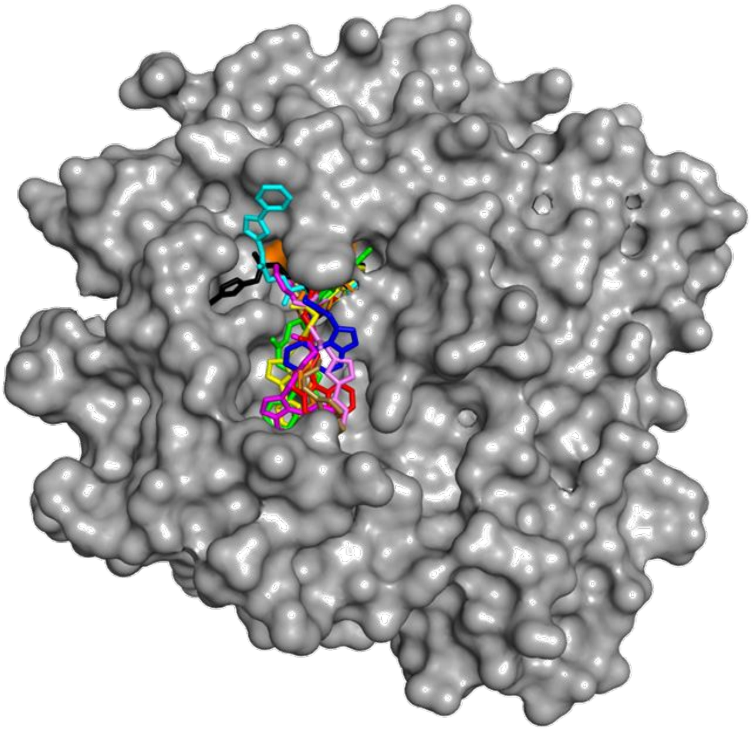

Figure S3. Ten unique 1771 docked poses prior to MD simulation. The top-scoring poses of 1771 (stick format) would be subjected to a 100-ns MD simulation to assess the stability of their respective binding mode in the eLtaS active site. The eLtaS protein is shown in grey surface representation. His347 and Arg356 are coloured in orange. The poses are assigned with the colour scheme: 1, black; 2, red; 3, green; 4, blue; 5, orange; 6, magenta; 7, brown; 8, violet; 9, cyan and 10, yellow.

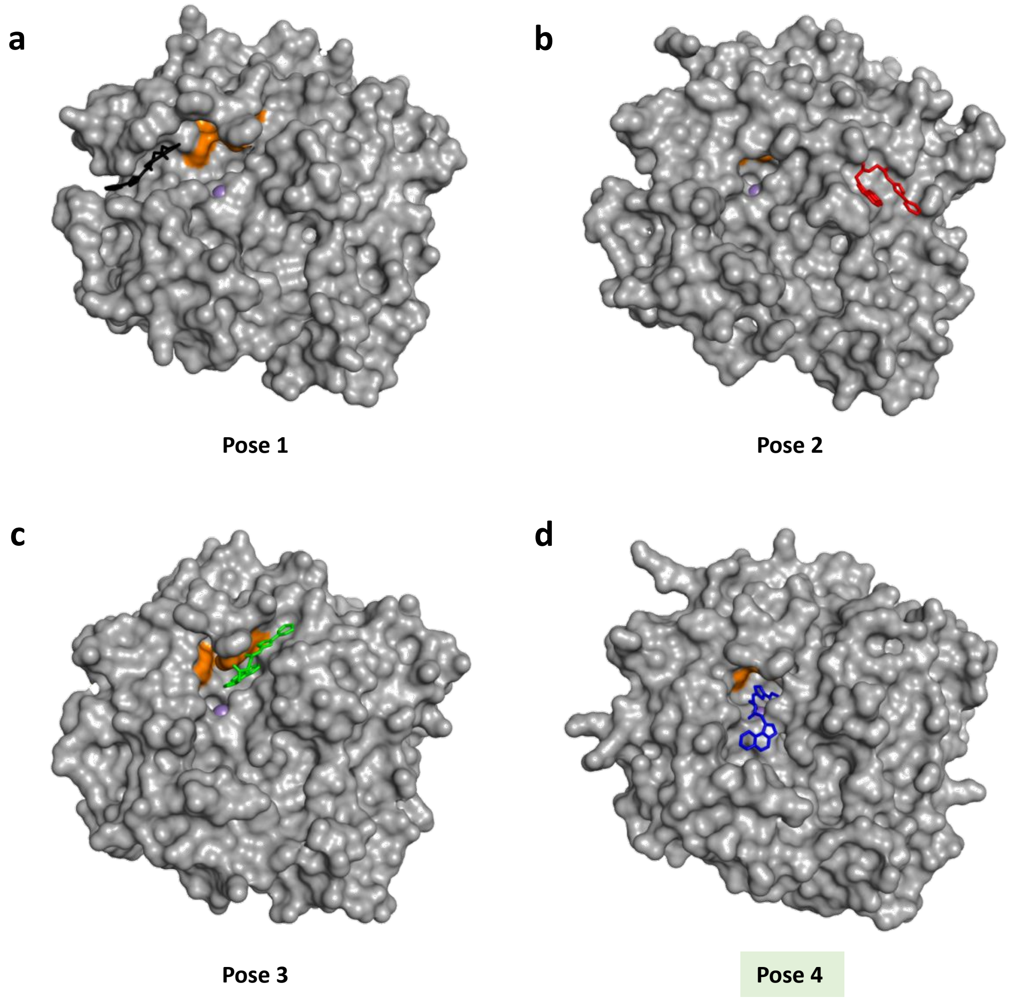

Figure S4. Final snapshots of the ten 1771 poses after the 100-ns MD simulation. The ten 1771 docked poses were subjected to a 100-ns MD simulation. Each image panel shows the final binding pose of 1771 (stick format) after the simulation. The eLtaS protein is shown in grey surface representation. His347 and Arg356 are coloured in orange. The Mn^{2+} ion is shown as a purple sphere. Pose **4** (green box) is selected as the most plausible binding pose of 1771 in the eLtaS active site. The poses are assigned with the colour scheme: 1, black; 2, red; 3, green; 4, blue; 5, orange; 6, magenta; 7, brown; 8, violet; 9, cyan and 10, yellow.

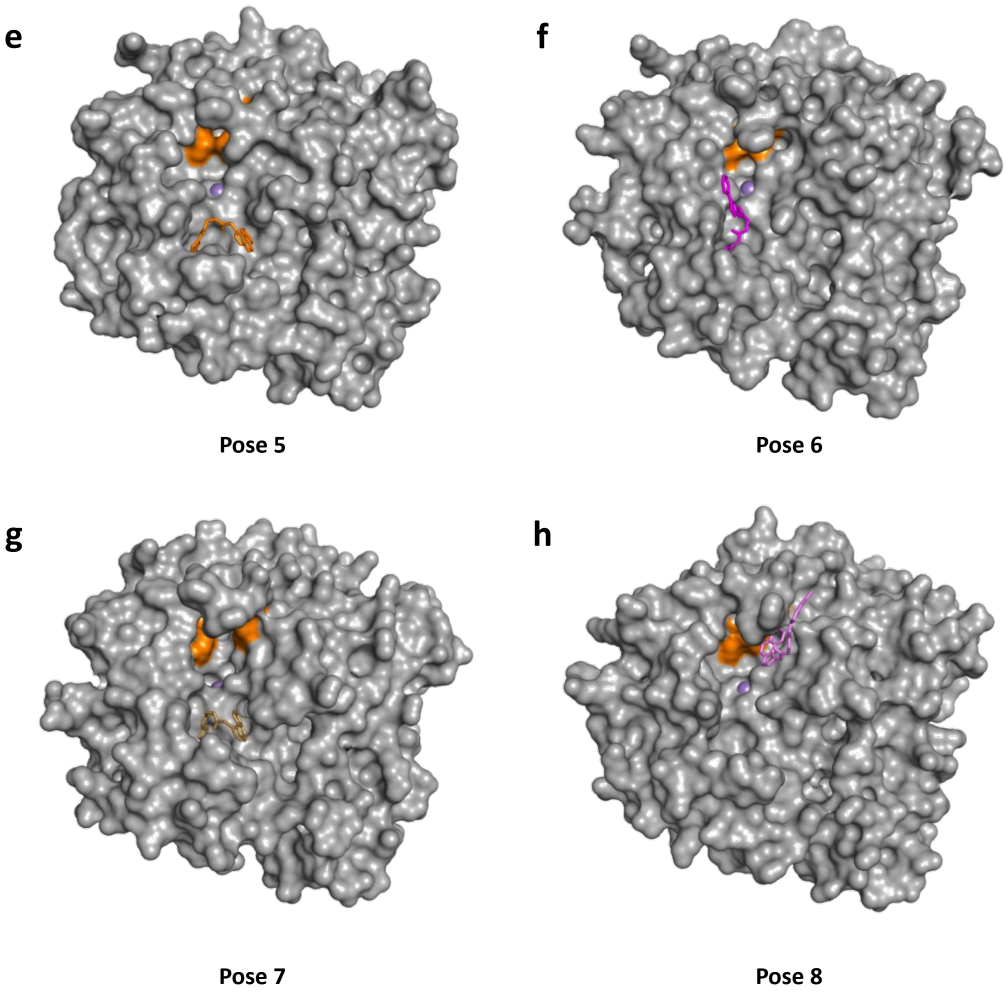

Figure S4 (continued). Final snapshots of the ten 1771 poses after the 100-ns MD simulation. The ten 1771 docked poses were subjected to a 100-ns MD simulation. Each image panel shows the final binding pose of 1771 (stick format) after the simulation. The eLtaS protein is shown in grey representation. His347 and Arg356 are coloured in orange. The Mn^{2+} ion is shown as a purple sphere. The poses are assigned with the colour scheme: 1, black; 2, red; 3, green; 4, blue; 5, orange; 6, magenta; 7, brown; 8, violet; 9, cyan and 10, yellow.

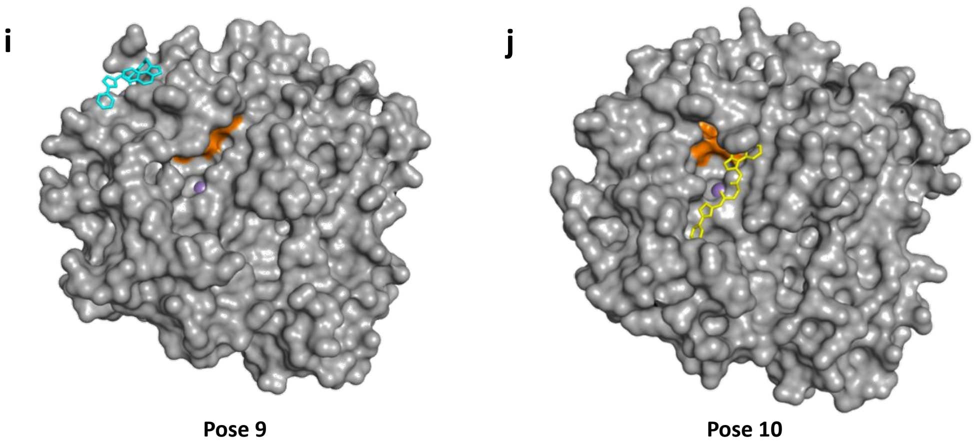

Figure S4 (continued). Final snapshots of the ten 1771 poses after the 100-ns MD simulation. The ten 1771 docked poses were subjected to a 100-ns MD simulation. Each image panel shows the final binding pose of 1771 (stick format) after the simulation. The eLtaS protein is shown in grey representation. His347 and Arg356 are coloured in orange. The Mn^{2+} catalytic ion is shown as a purple sphere. The poses are assigned with the colour scheme: 1, black; 2, red; 3, green; 4, blue; 5, orange; 6, magenta; 7, brown; 8, violet; 9, cyan and 10, yellow.

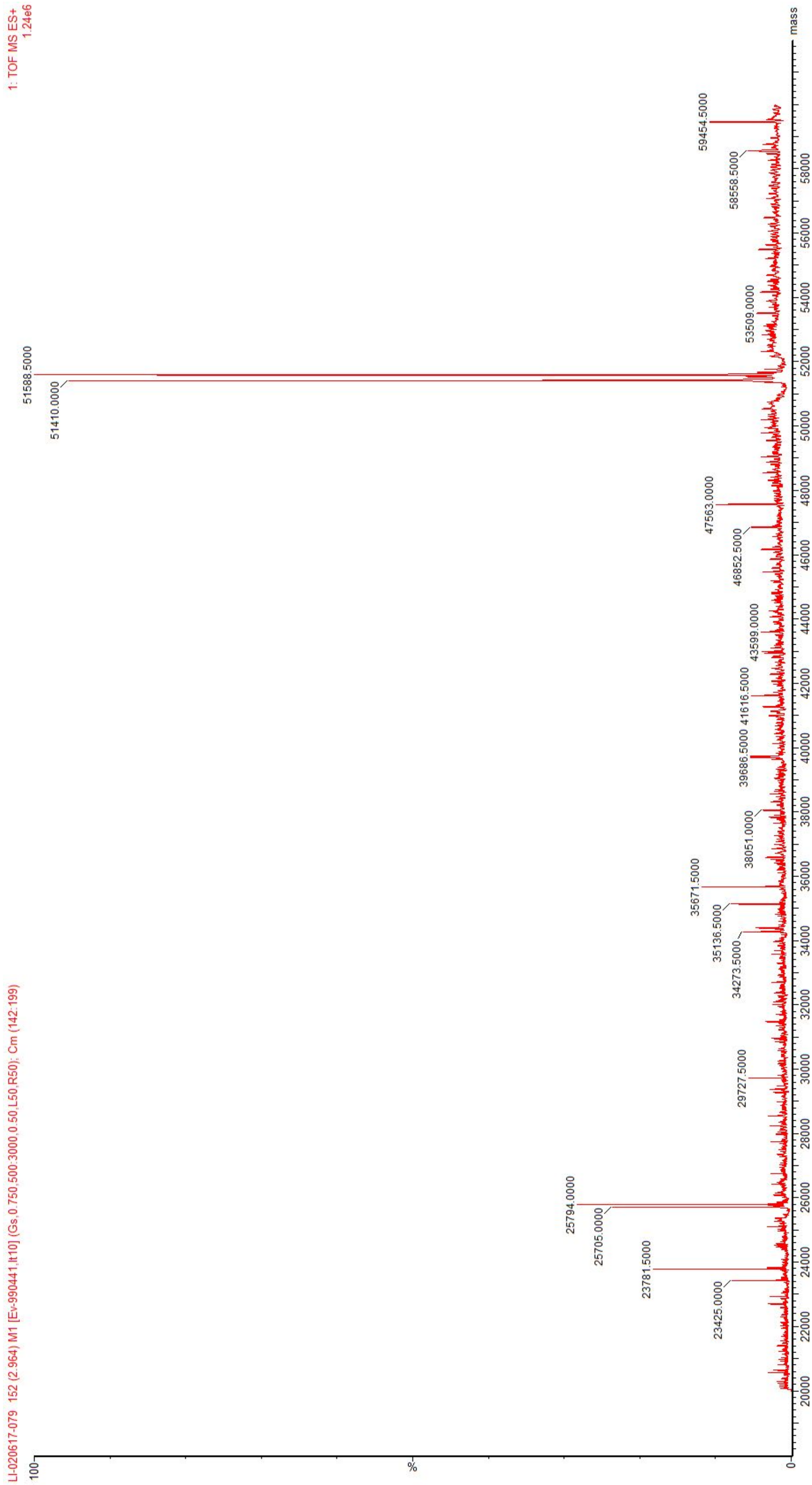

Figure S5. LC-MS/MS Spectra of His416Ala mutant eLtaS

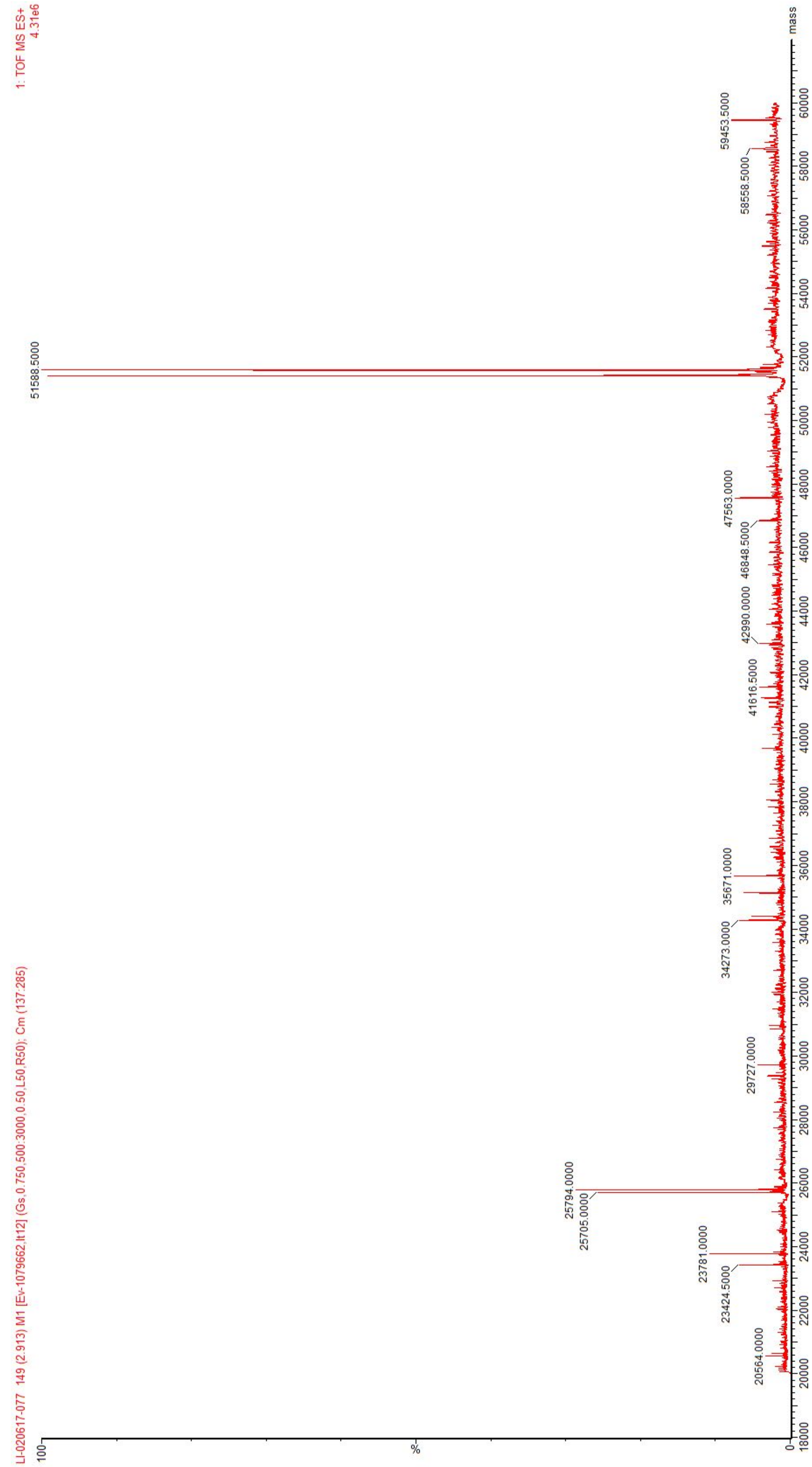

Figure S6. LC-MS/MS Spectra of His347Ala mutant eLtaS

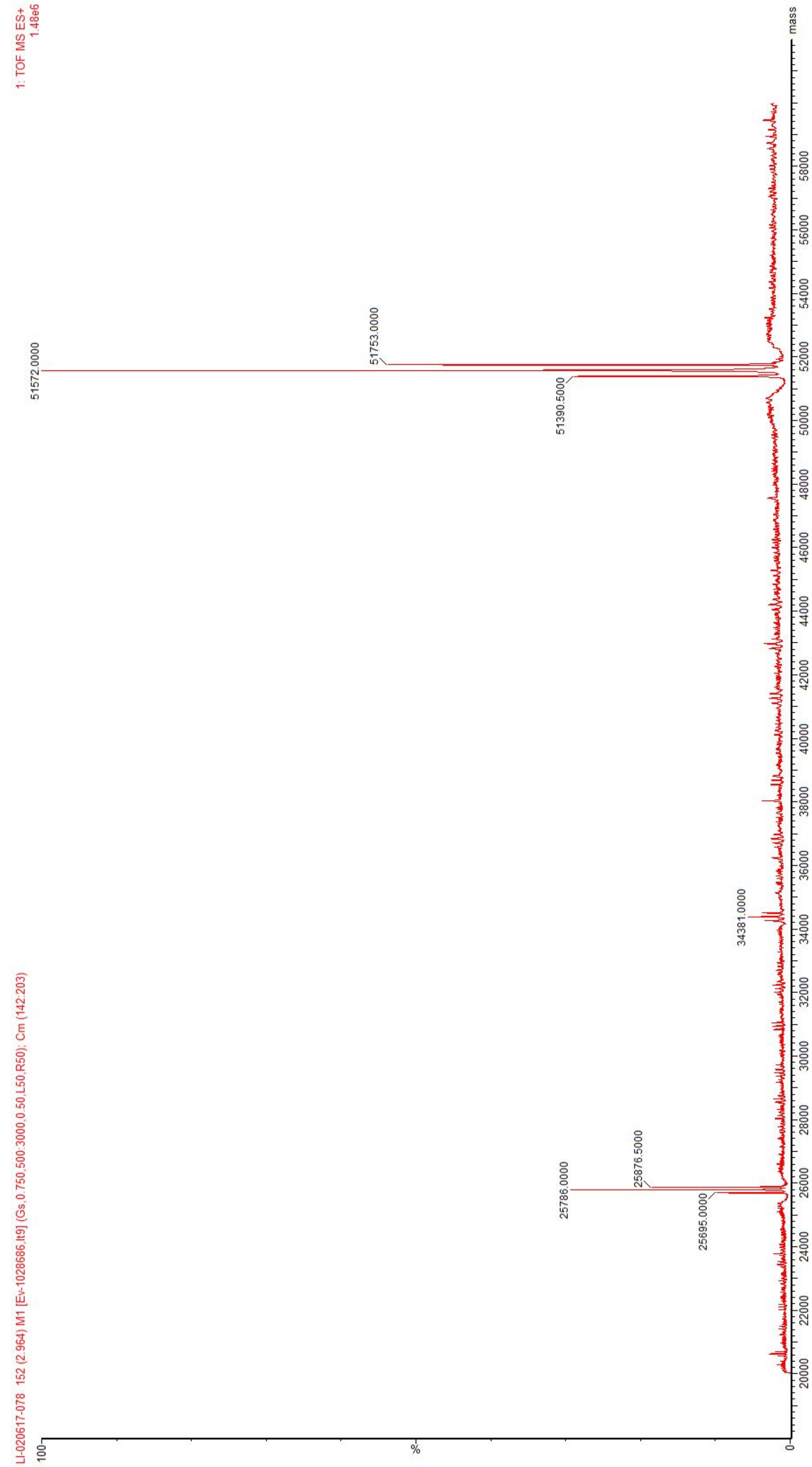

Figure S7. LC-MS/MS Spectra of Arg356Ala mutant eLtaS

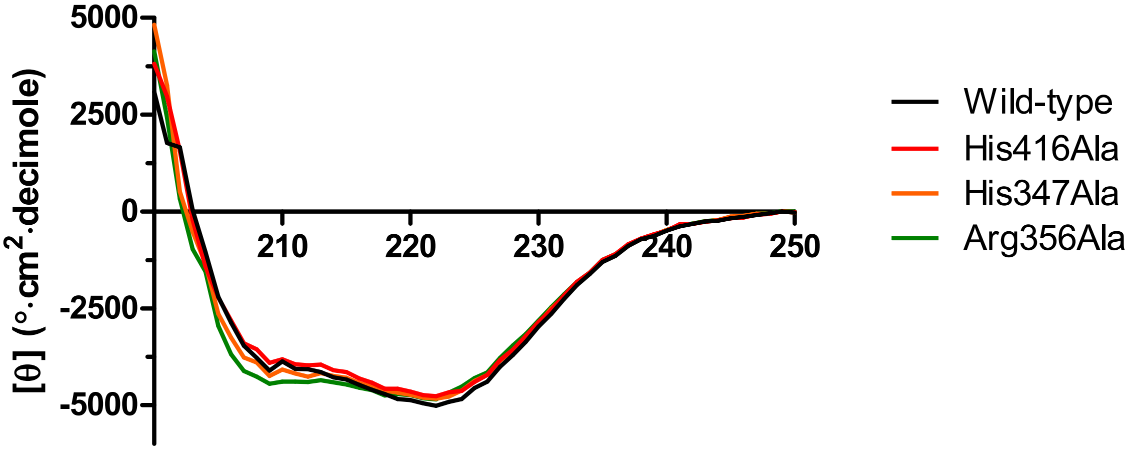

Figure S8. Circular dichroism spectra of wild-type eLtaS with the His416Ala, His347Ala and Arg356Ala mutants.

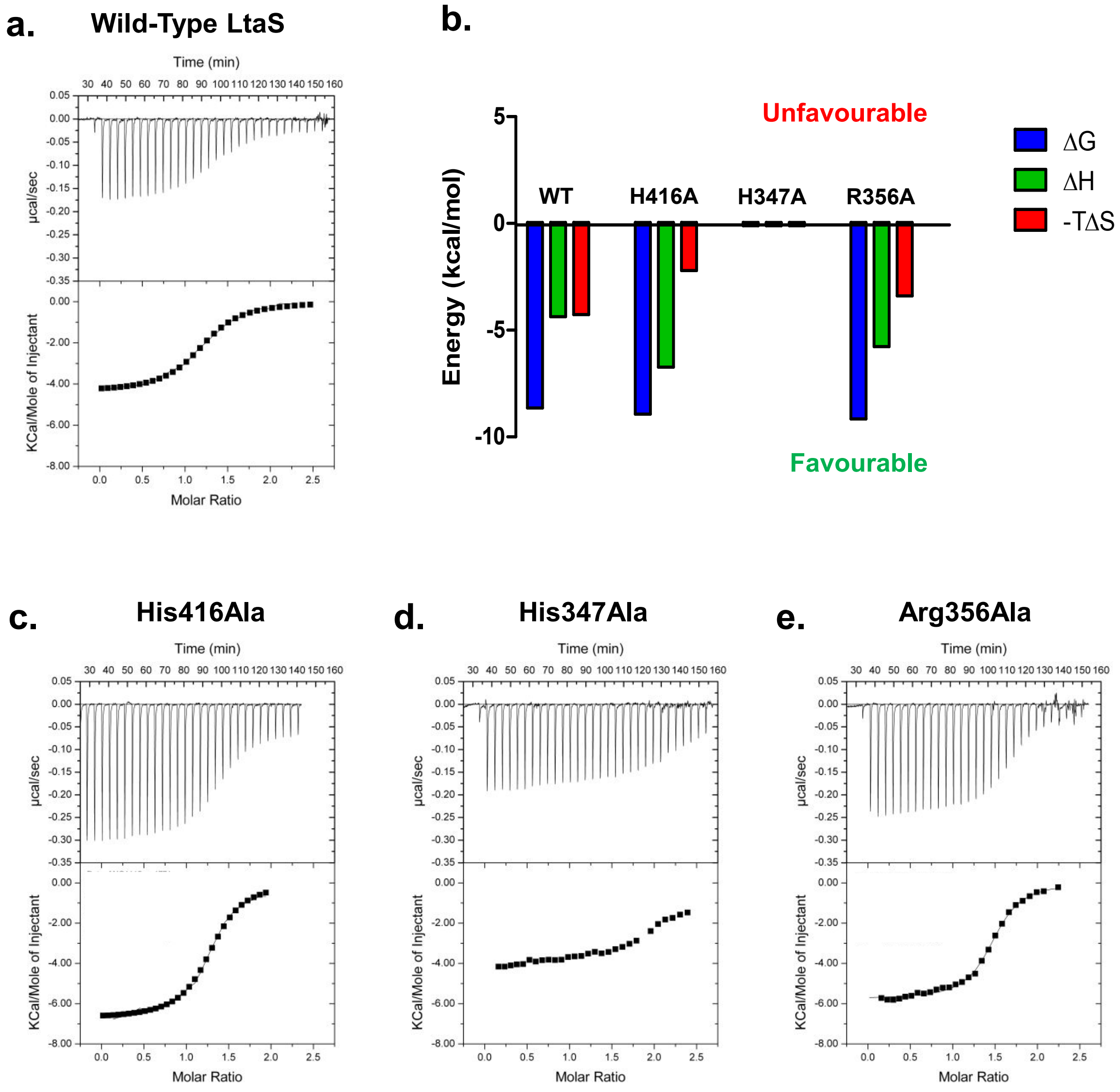

Figure S9. Alanine-mutagenesis of the eLtaS active site residues. (**a**) ITC analysis of 1771 binding to wild-type eLtaS. (**b**) Thermodynamic signatures of 1771 binding to WT or mutated eLtaS. The Gibbs free energy of binding (ΔG; blue), enthalpy (ΔH; green) and entropy (ΔS; red) are shown as bars in the histogram. The same ITC method was used to assess the binding of 1771 to eLtaS with mutations (**c**) His416Ala (**d**) His347Ala and (**e**) Arg356Ala. Top panel of each thermogram depicts the raw calorimetric titration profile. Bottom panel shows the fitting of the experimental heat of binding to the model equations to derive the thermodynamic signatures.

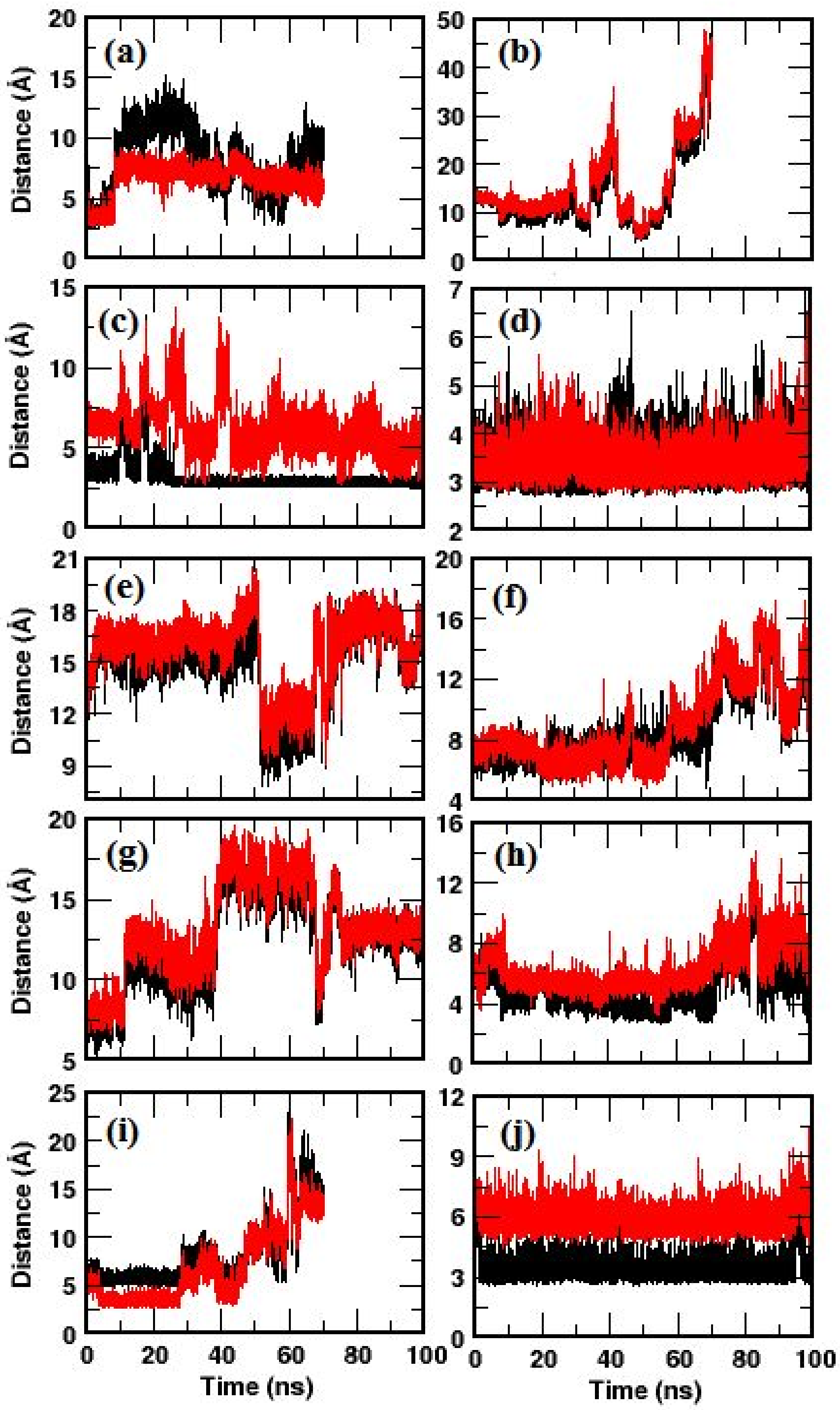

Figure S10. **Interactions between the oxadiazole nitrogen atoms of 1771 with residues His347 and Arg356 along the simulation time.** Each of the selected ten poses from ensemble docking was subjected to a 100-ns MD simulation. (**a-j**) The distances (Å) between the oxadiazole nitrogen atoms of the ten poses and His347 and Arg356 during the MD simulation. The hydrogen bond donor-acceptor distance is taken to be about 3Å. Red and black lines represent the distance for His347--1771 and Arg356--1771, respectively.

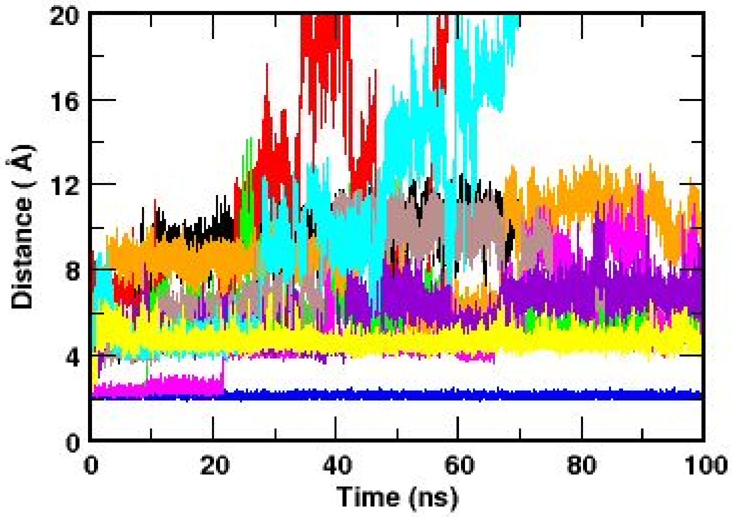

Figure S11. **Interaction between the amide oxygen atom of 1771 with the Mn^{2+} ion along the simulation time.** Each of the selected ten poses from the ensemble docking was subjected to a 100-ns MD simulation. The graph shows the distance (Å) between the oxygen atom of the ten poses and the Mn^{2+} ion during the MD simulation. The poses are assigned with the colour scheme: 1, black; 2, red; 3, green; 4, blue; 5, orange; 6, magenta; 7, brown; 8, violet; 9, cyan and 10, yellow.

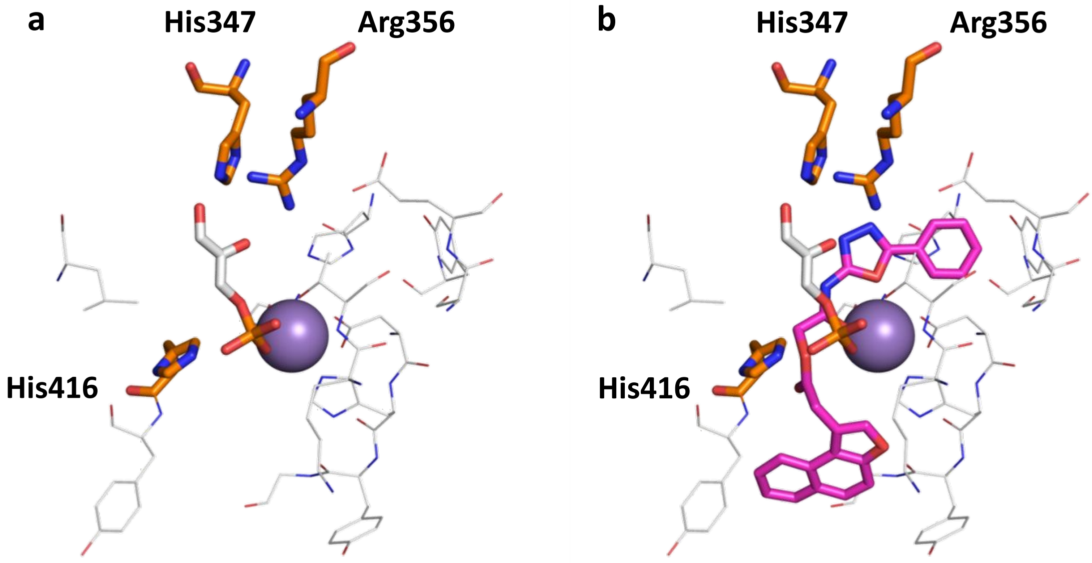

Figure S12. **Binding poses of GroP and 1771 in eLtaS.** (**a**) Residues of eLtaS active site are shown with a bound GP9 (grey stick) at the catalytic centre (PDB-ID 2W5T). (**b**) Overlay of the 1771 binding pose (magenta stick) at the active site. All residues of the eLtaS active site within 4Å of 1771 are shown in gray line format. Hydrogen-bonding residues are shown in orange stick format. The Mn^{2+} ion is shown as a purple sphere.

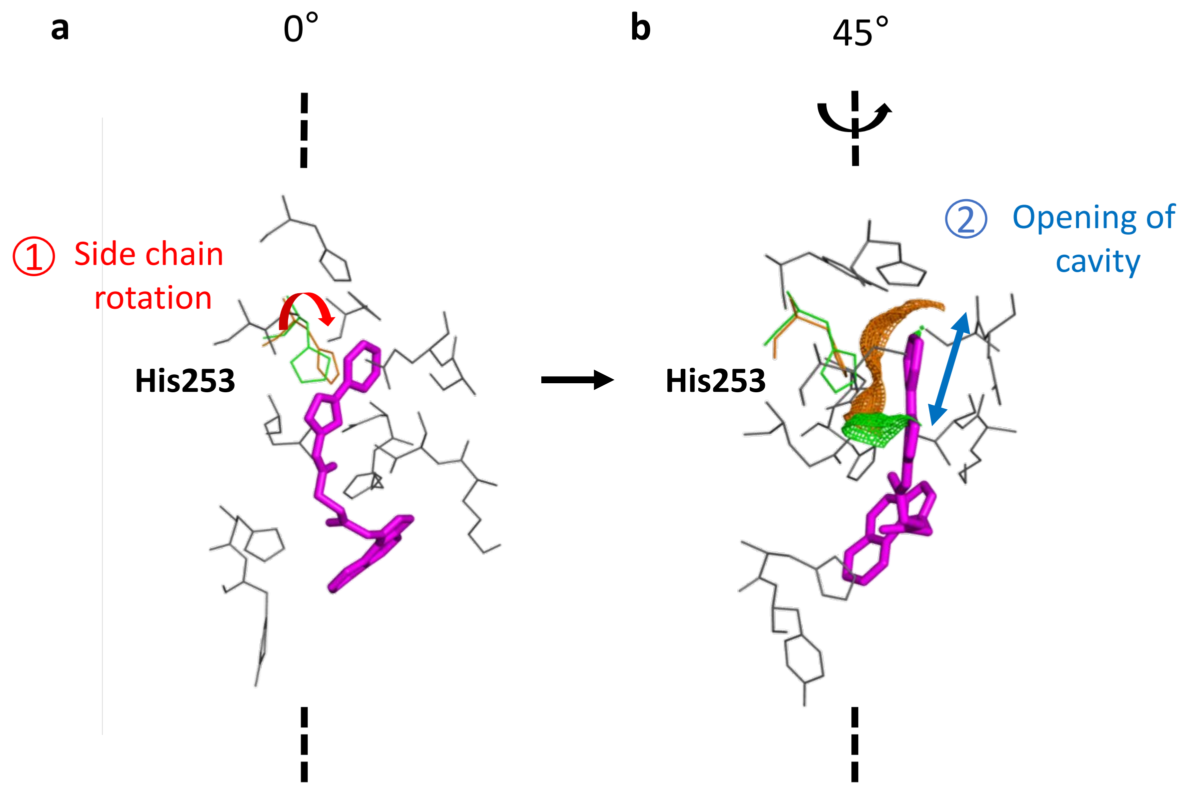

Figure S13. **Conformational rotation of His253 opens up a new sub-pocket for binding.** (**a**) Initial (green line) and final (orange line) positions of His253 after a shift in side-chain romateric state (red arrow) upon ligand binding. (**b**) A rotated view of the eLtaS active site showing the opening of the sub-pocket (blue up down arrow). All residues of the eLtaS active site within 4Å of 1771 are shown in grey line format. Compound 1771 is shown in magenta stick format. The molecular surface of His253 before (green) and after (orange) conformational rotation shown in mesh representation.

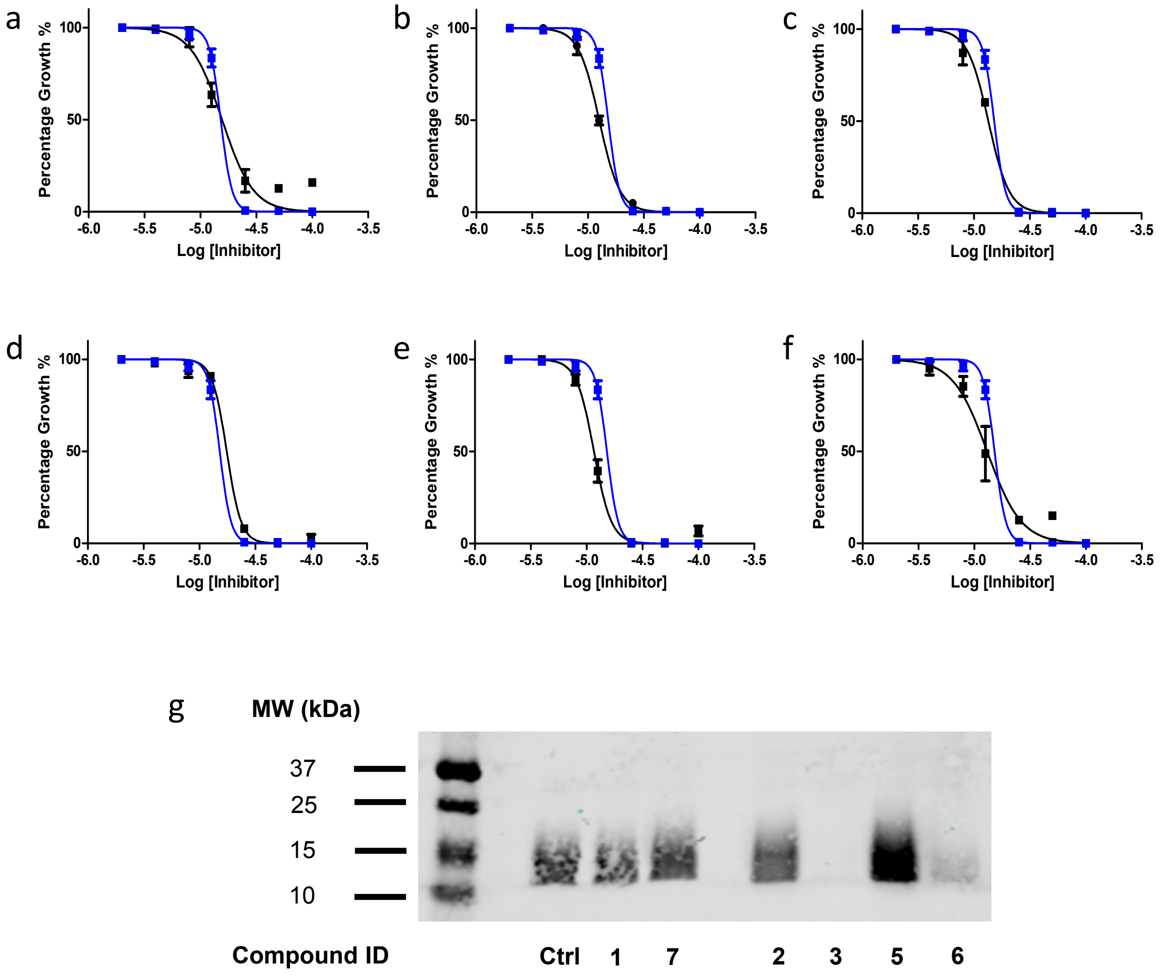

Figure S14. **Shortlisted candidates as potential eLtaS inhibitors.** These compounds were selected on the basis that their antimicrobial potencies, which are similar or better than 1771. Dose-response curves (black line) show the inhibitory effects of compounds (**a**) **1**, (**b**) **2**, (**c**) **3**, (**d**) **5**, (**e**) **6** and (**f**) **7** on the growth of *S. aureus*. Dose-response curve of 1771 (blue line) is shown for reference. The curve for compound **4** is shown elsewhere in the main text. The data represent the mean $\pm$ s.e.m. of n = 3 independent experiments, each performed in a duplicate. (**g**) Immunoblotting of LTA in *S. aureus* treated with 10 μM of the indicated compounds. The data shown is a representative of two independent western blot experiments.

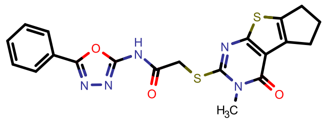

1
Z1674607

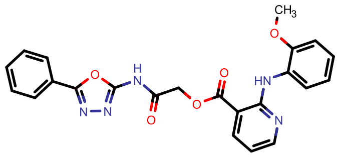

2
Z15691256

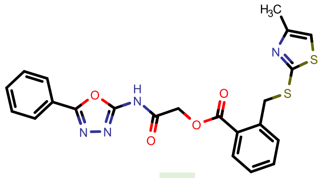

3
Z17116140

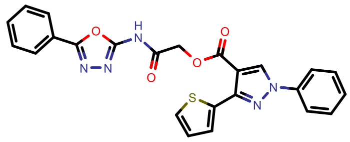

4
Z18903036

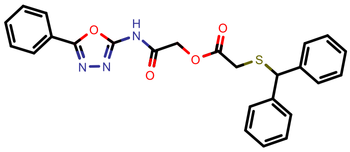

5
Z17092837

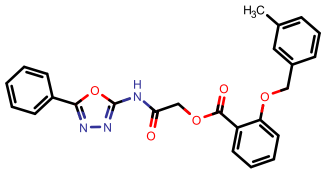

6
Z17366769

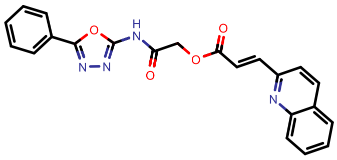

7
Z25344027

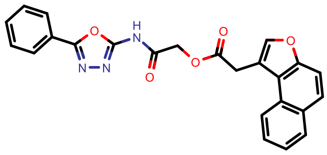

1771
Z25275760

Figure S15. **Chemical structures of shortlisted candidates as potential eLtaS inhibitors.** Chemical structures of the compounds evaluated for LTA synthesis inhibition are shown above. Compounds that showed LTA inhibition is highlighted in green. Compound 1771 discovered by Richter *et al.* is highlighted in blue. The Enamine catalog ID for each compound is listed as shown

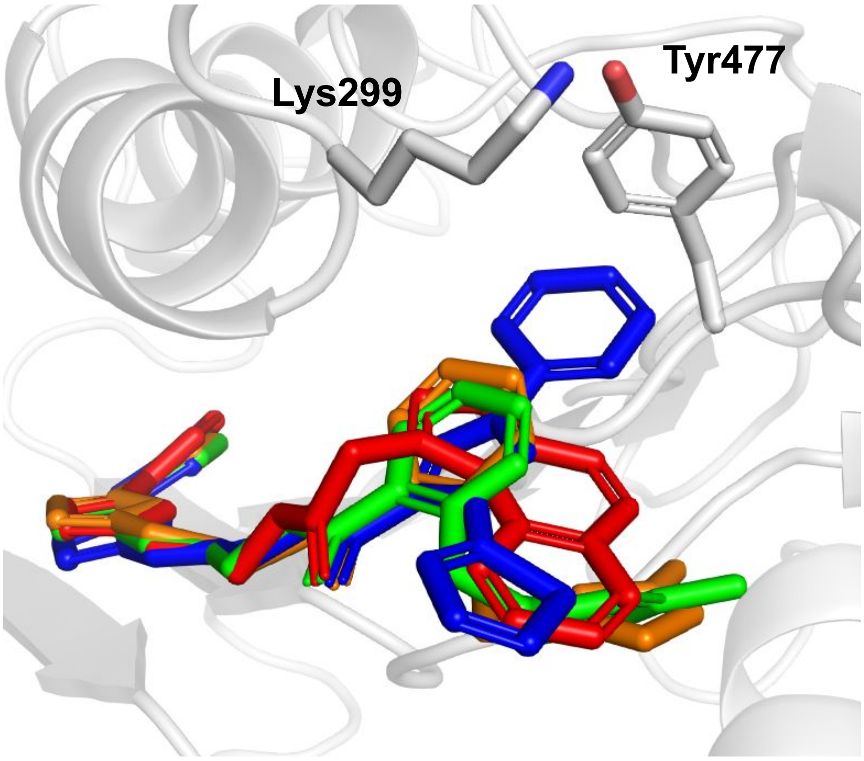

Figure S16. **Docked position of compound 3, 4, 6 and 1771.** The docked position of compounds **3**, **4**, **6** and **1771** are shown relative to the binding cavity lined by residues Lys299 and Tyr477. Compounds **3**, **4**, **6** and **1771** shown as orange, blue, green and red, respectively.

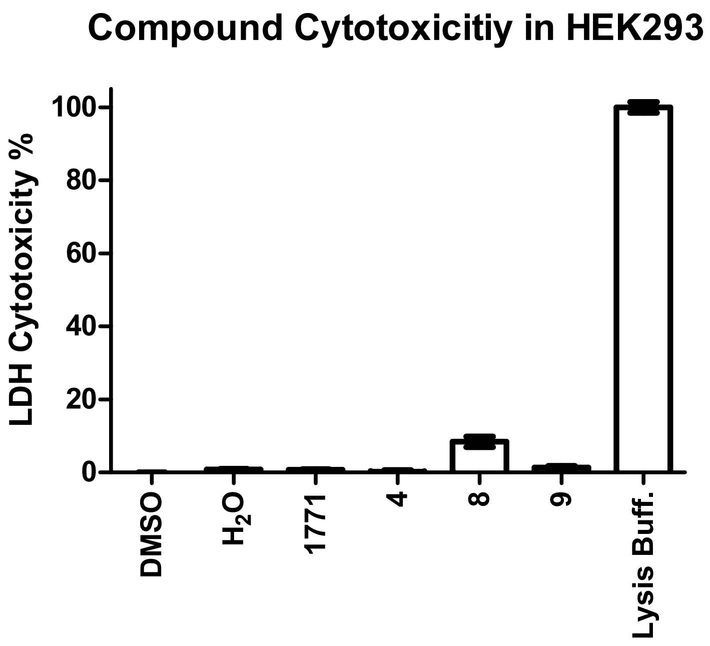

Figure S17. **Cytotoxicity assessment of compounds.** Cytotoxicity of the compounds was evaluated using lactate-dehydrogenase (LDH) assays. The mammalian HEK293 cells were treated with DMSO solvent, water, the indicated compounds at a concentration of 200 μM for 24 hrs or with lysis buffer . The data shown are the mean percentage $\pm$ s.e.m. of n = 4 independent experiments.

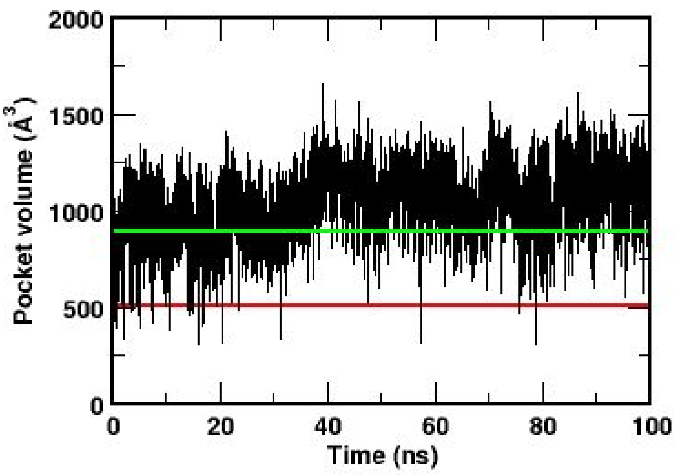

Figure S18. **Dynamic Nature of the eLtaS Active Site.** The black line shows the changing of the active site pocket volume of the apo eLtaS across the simulation time-course. The conformations of the eLtaS crystal structure (PDB ID 2W5T and 2W5Q) were also visited in the simulation. The pocket volumes of 2W5Q (503 Å^3; red line) and 2W5T (889 Å^3; green line) are plotted on the figure.

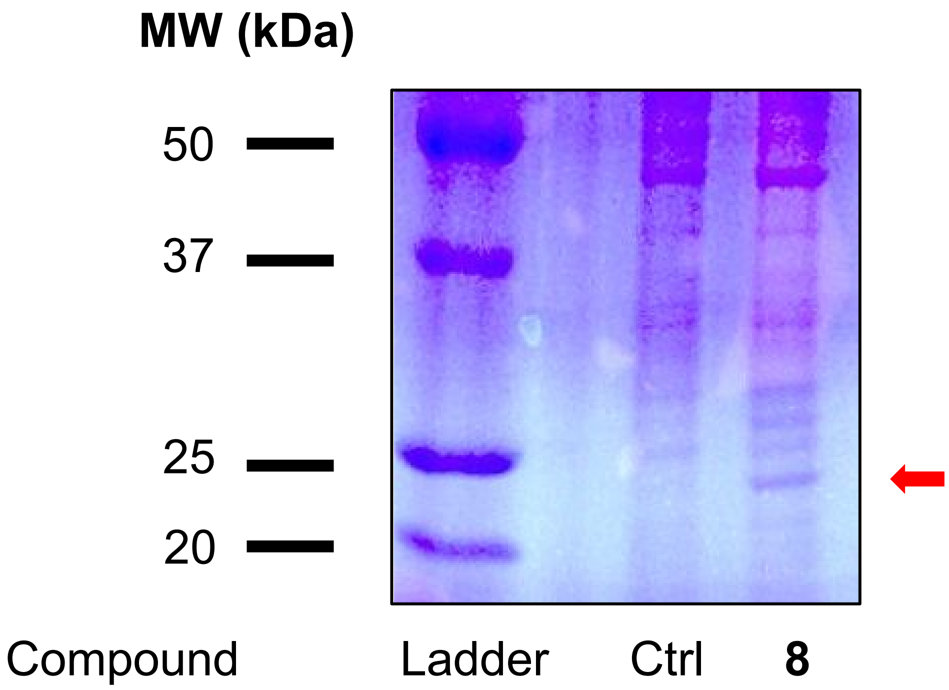

Figure S19. **Up-regulation of the lytic transglycosylase IsaA compound 8.** Cell extracts were isolated from *S. aureus* that were treated with or without 100 μM of compound **8**. The proteins in these extracts are separated and analyzed by SDS-PAGE. The gel shown is representative across three independent experiments (two of these gels were sent for protein identification using LC/MS-MS).

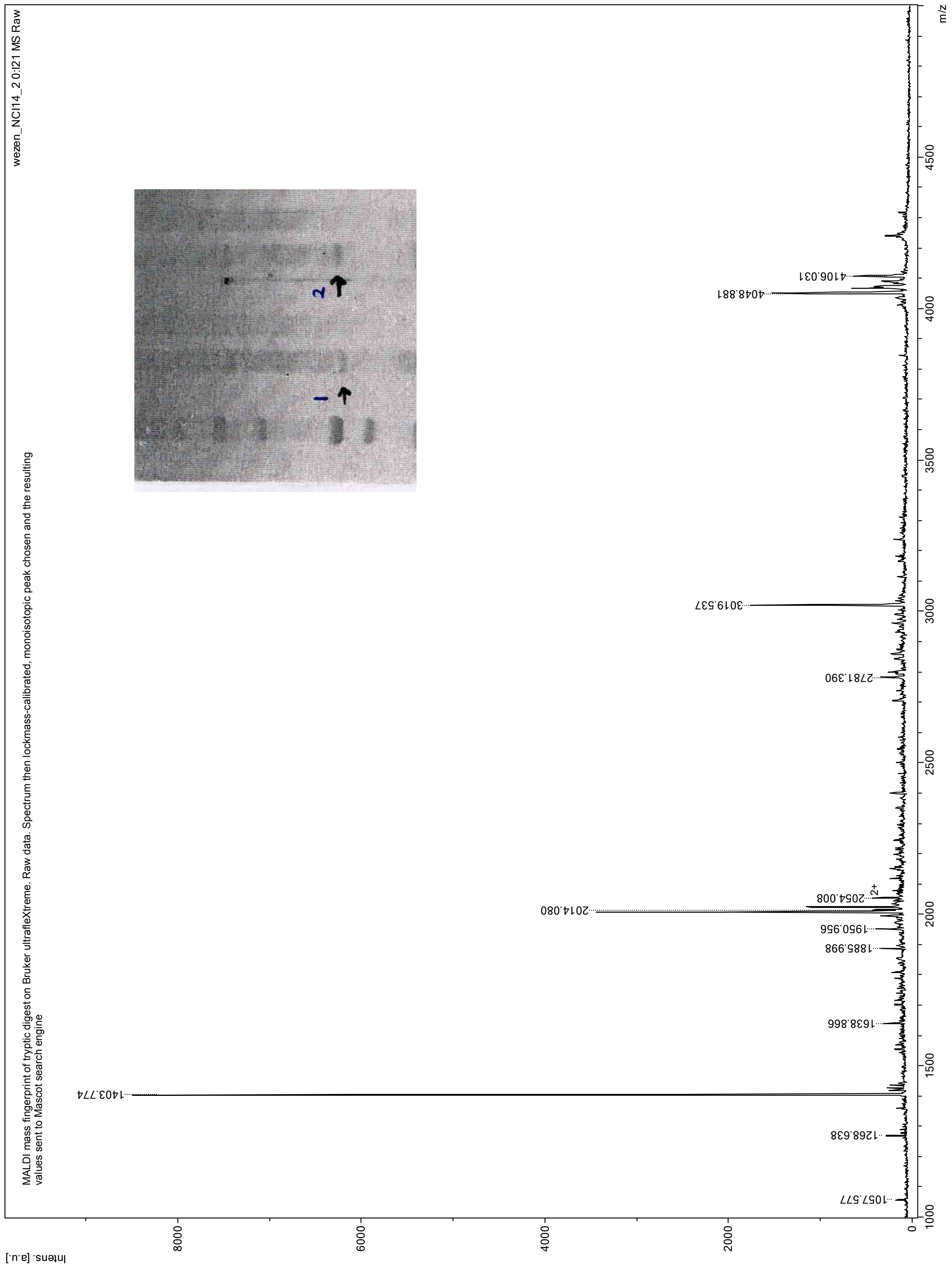

Figure S20. **Identification of the anomalous protein using LC-MS/MS.**

```
Search title    : wezen_NCI14_1
MS data file    : wezen_NCI14_1.mgf
Database        : NCBIprot 20161127 (106762850 sequences; 39119668168 residues)
Taxonomy        : Bacteria (Eubacteria) (77258759 sequences)
Timestamp       : 2 Dec 2016 at 11:52:27 GMT
Top Score       : 340 for KDS65301.1, transglycosylase, partial [Staphylococcus aureus]
```

Mascot Score Histogram

Protein score is -10*Log(P), where P is the probability that the observed match is a random event.
Protein scores greater than 91 are significant (p<0.05).
Protein scores are derived from ions scores as a non-probabilistic basis for ranking protein hits.

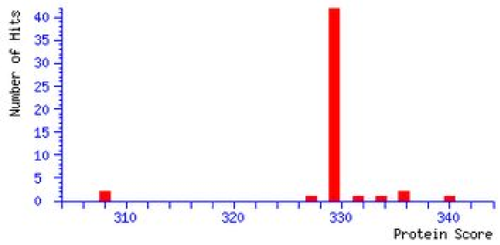

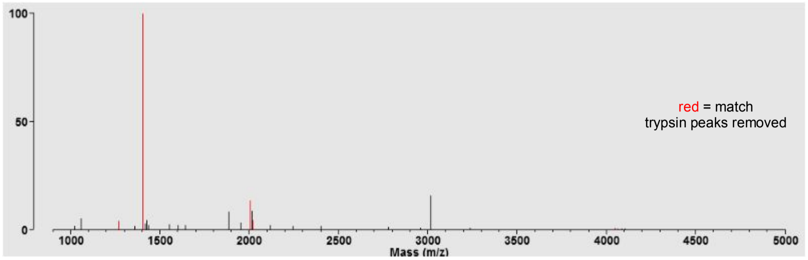


```
  1 AGADYTTSYN QGSNVQSVSY NAQSSNSNVE AVSAPTYHNY STSTTSSSVR
 51 LSNGNTAGAT GSSAAQIMAQ RTGVSASTWA AIIARESNGQ VNAYNPSGAS
101 GLFQTMPGWG PTNTVDQQIN AAVKAYKAQG LGAWGF
```

Will not see signals from 4-147 residues as only 2 Lys and they are followed by Asp = very restricted cleavage, so no peptides produced within dynamic range of analysis

```
        10         20         30         40         50
MKKTI MASSL AVALG VIGYA AGTGH QAHAA EVNVD QAHLV DLAHN HQDQL
        60         70         80         90        100
NAAPI KDGAY DIHFV KDGFQ YNFTS NGTTW SWSYE AANGQ TAGFS NVAGA
       110        120        130        140        150
DYTTS YNQGS DVQSV SYNAQ SSNSN VEAVS APTYH NYSTS TTSSS VRLSN
       160        170        180        190        200
GNTAG ATGSS AAQIM AQRTG VSAST WAAII ARESN GQVNA YNPSG ASGLF
       210        220        230
QTMPG WGPTN TVDQQ INAAV KAYKA QGLGA WGF
```

	Accession	Mass	Score	Description
1.	KDS65301.1	13890	340	transglycosylase, partial [Staphylococcus aureus]
2.	WP_041497937.1	18251	335	transglycosylase, partial [Staphylococcus aureus]
3.	WP_044167235.1	18762	335	transglycosylase, partial [Staphylococcus aureus]
4.	WP_049882378.1	21295	333	transglycosylase, partial [Staphylococcus aureus]
5.	WP_000168596.1	23587	331	hypothetical protein [Staphylococcus aureus]
6.	WP_000751262.1	24201	330	transglycosylase [Staphylococcus aureus]

```
  1 MKKTIMASSL AVALGVIGYA AGTGHQAHAA EVNVDQAHLV DLAHNHQDQL
 51 NAAPIKDGAY DIHFVKDGFQ YNFTSNGTTW SWSYEAANGQ TAGFSNVAGA
101 DYTTSYNQGS DVQSVSYNAQ SSNSNVEAVS APTYHNYSTS TTSSSVRLSN
151 GNTAGATGSS AAQIMAQRTG VSASTWAAII ARESNGQVNA YNPSGASGLF
201 QTMPGWGPTN TVDQQINAAV KAYKAQGLGA WGF
```

1. KDS65301.1 Mass: 13890 Score: 340 Expect: 7.7e-27 Matches: 6
transglycosylase, partial [Staphylococcus aureus]

Observed	Mr(expt)	Mr(calc)	ppm	Start	End	Miss	Ions	Peptide
1268.6346	1267.6274	1267.6349	-5.99	125	136	1	68	K.AYKAQGLGAWGF.-
1403.7623	1402.7550	1402.7569	-1.30	72	85	0	102	R.TGVSASTWAAIIAR.E
2005.9699	2004.9626	2004.9647	-1.03	51	71	0	107	R.LSNGNTAGATGSSAAQIMAQR.T
2021.9683	2020.9610	2020.9596	0.71	51	71	0	---	R.LSNGNTAGATGSSAAQIMAQR.T + Oxidation (M)
4048.8926	4047.8854	4047.9021	-4.14	86	124	0	---	R.ESNGQVNAYNPSGASGLFQTMPGWGPTNTVDQQINAAVK.A
4064.9149	4063.9076	4063.8970	2.60	86	124	0	---	R.ESNGQVNAYNPSGASGLFQTMPGWGPTNTVDQQINAAVK.A + Oxidation (M)

Figure S20 (continued).Identification of the anomalous protein using LC-MS/MS.

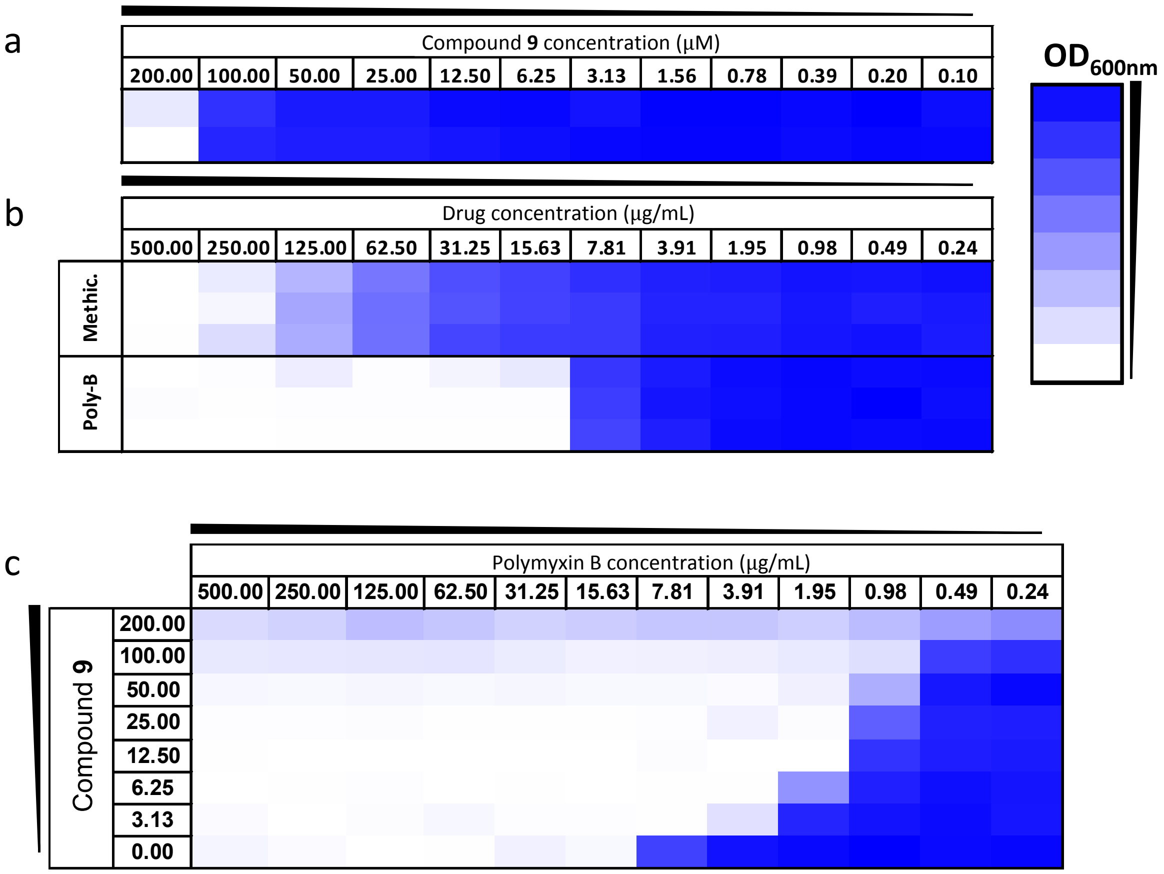

Figure S21. **Potentiation of Polymyxin B by compound 9 against MRSA.** The figure above shows the growth inhibitory effects of (**a**) compound **9**, (**b**) methicillin and Polymyxin B when used against MRSA alone. (**c**) The synergistic effect of Polymyxin B and compound **9** against MRSA is assessed using a micro-dilution checkerboard analysis. Percentage growth is shown as a heat plot. Three independent experiments were replicated.

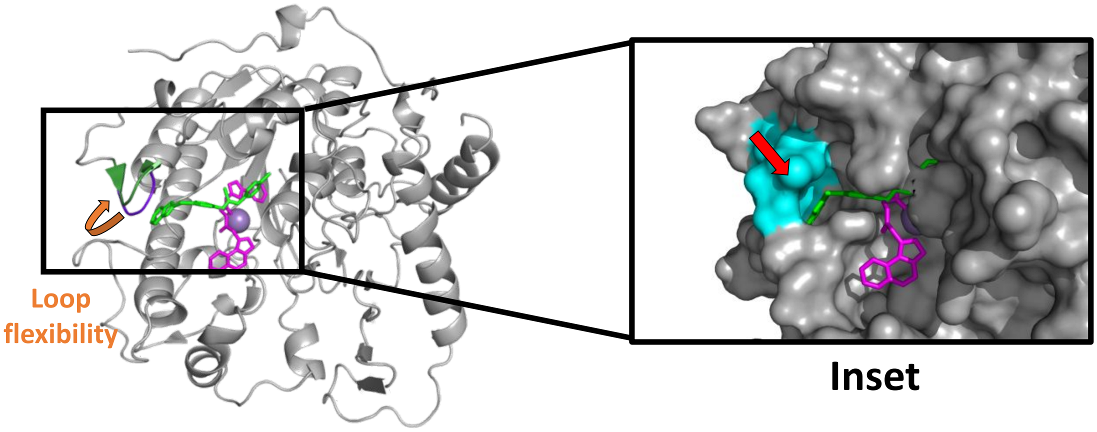

Figure S22. **Predicted binding pose of compounds 1771 and compound 9.** The figure above shows the conformational change of a loop that formed a transient cryptic pocket. Compounds 1771 and compound **9** are shown in magenta and green stick format, respectively. The active site Mn^{2+} shown as purple sphere

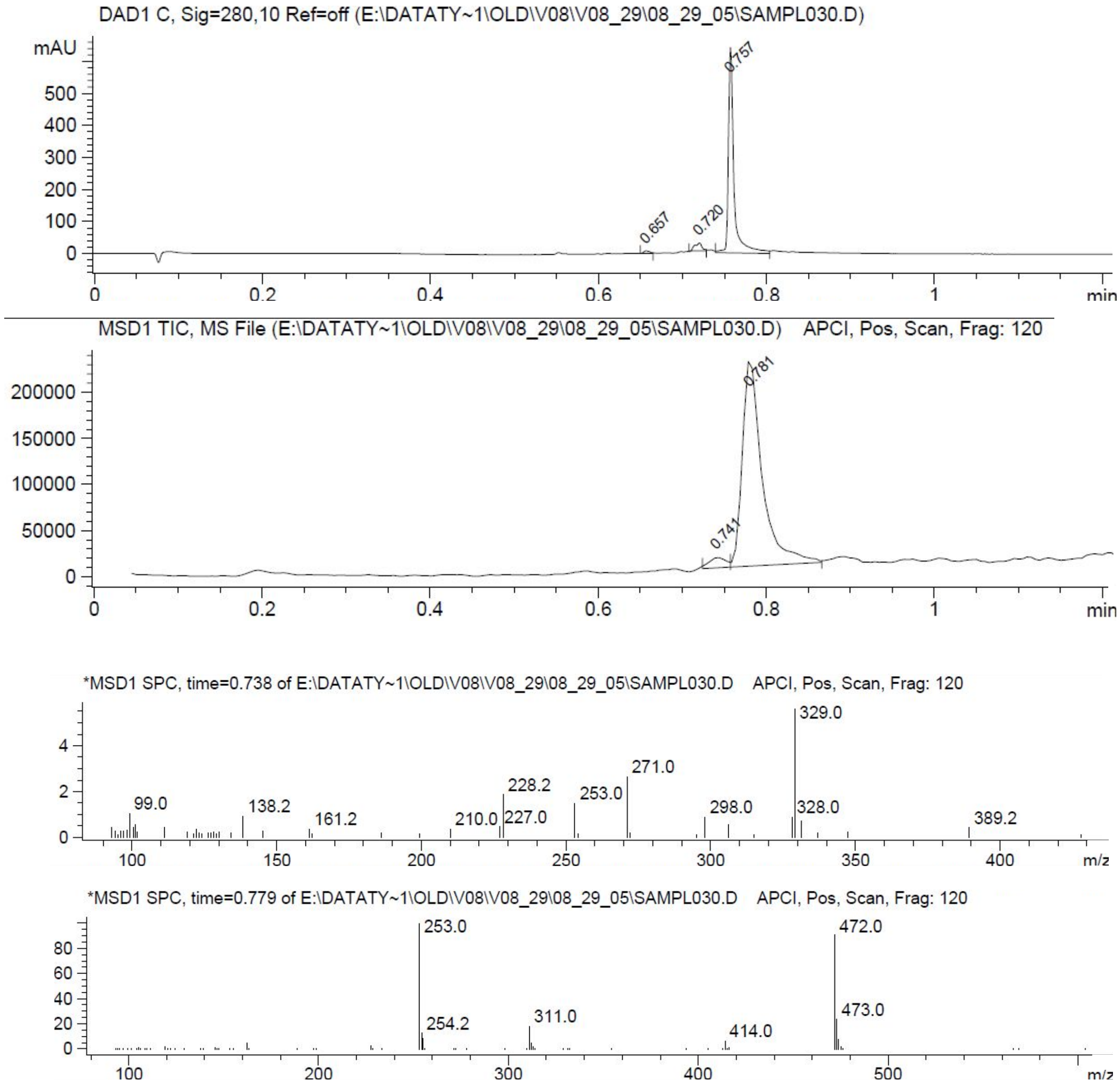

Figure S23. **Purity data of compound 4.** The figure above shows the Liquid Chromatography – Mass Spectrometry (LC-MS) data of compound **4**. Data courtesy of the chemical vendor Enamine Ltd.

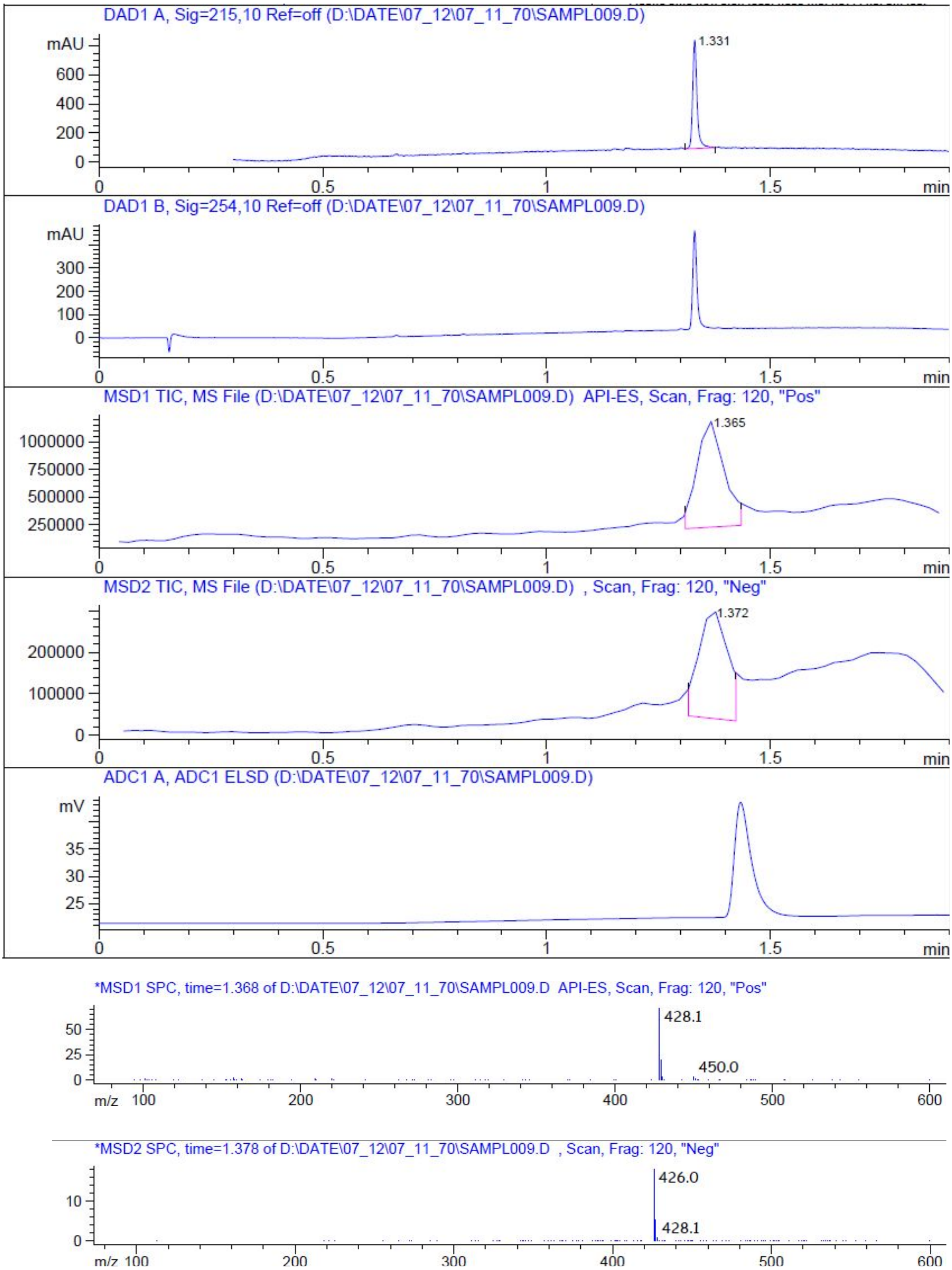

Figure S24. **Purity data of compound 1771.** The figure above shows the Liquid Chromatography – Mass Spectrometry (LC-MS) data of compound 1771. Data courtesy of the chemical vendor Enamine Ltd.

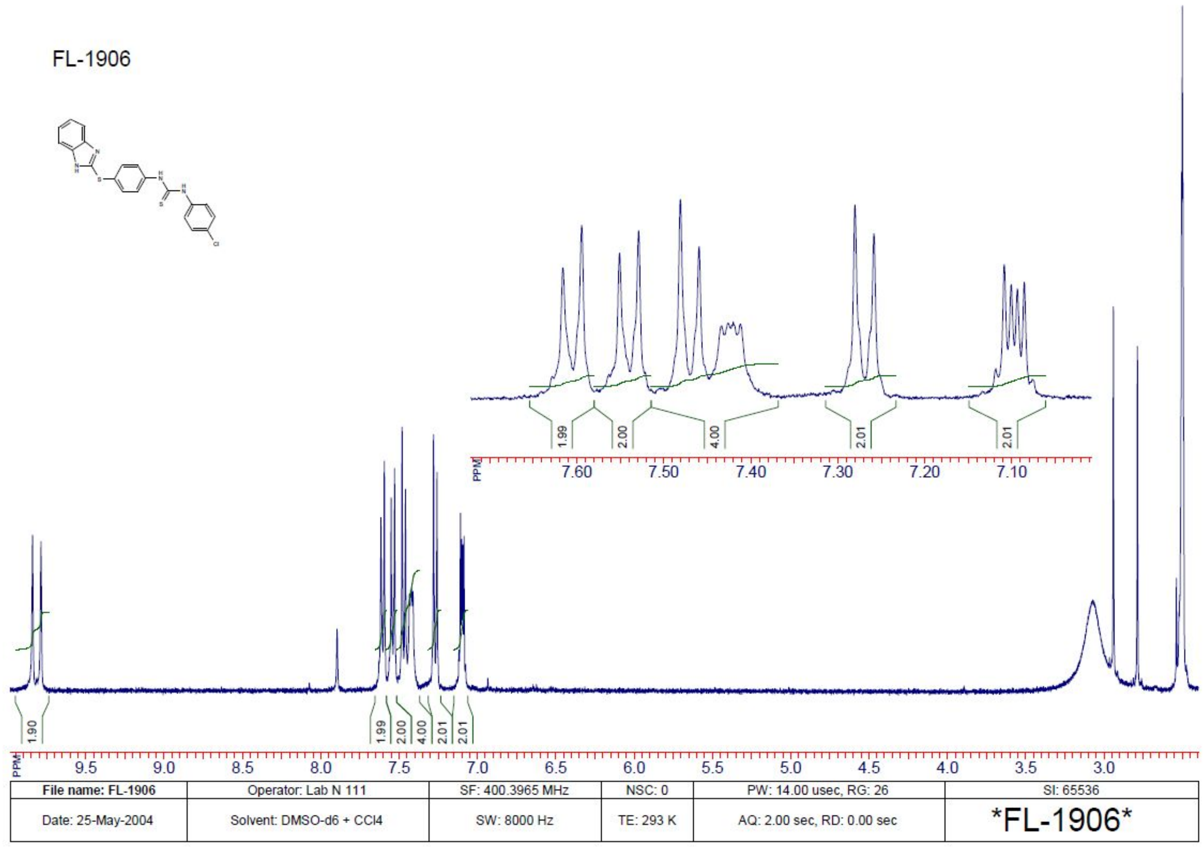

Figure S25. **Purity data of compound 9.** The figure above shows the Nuclear Magnetic Resonance (NMR) spectrometry data of compound **9**. Data courtesy of the chemical vendor Enamine Ltd.

Table S1. **Docking population of each site by "blind" docking.** Table shows the number of times 1771 had been docked to a particular site in a conformer by Autodock 4. Each of the twenty conformers (including the apo crystal structure) was docked one hundred times; hence, yielding two thousand docked poses overall. The number of times 1771 was docked at a particular site were summed across all twenty conformers and then divided by two thousand. This is further expressed as a percentage of the docking population.

Conformers	Site A	Site B	Site C	Others
2W5T	49	6	31	14
1	50	50	0	0
2	50	41	1	8
3	51	42	4	3
4	96	1	1	2
5	94	0	5	1
6	69	28	0	3
7	66	31	2	1
8	53	38	1	8
9	93	6	0	1
10	57	35	0	8
11	86	9	0	5
12	49	50	0	1
13	55	39	6	0
14	32	68	0	0
15	64	35	0	1
16	92	4	4	0
17	92	6	0	2
18	71	25	0	4
19	31	65	0	4
**Total**	**1300**	**579**	**55**	**66**
**Percentage (%)**	**65.0**	**29.0**	**2.8**	**3.3**
